# Morbidity associated with *Schistosoma mansoni* infection in north-eastern Democratic Republic of the Congo

**DOI:** 10.1371/journal.pntd.0009375

**Published:** 2021-12-02

**Authors:** Maurice M. Nigo, Peter Odermatt, David Wully Nigo, Georgette B. Salieb-Beugelaar, Manuel Battegay, Patrick R. Hunziker

**Affiliations:** 1 Nanomedicine Translation Group, Medical Intensive Care Clinic, University Hospital Basel University of Basel, Basel, Switzerland; 2 CLINAM—European Foundation for Clinical Nanomedicine, Basel, Switzerland; 3 University of Basel, Basel, Switzerland; 4 Institut Supérieur des Techniques Médicales (ISTM) Nyankunde, Democratic Republic of Congo; 5 Swiss Tropical and Public Health Institute, Basel, Switzerland; 6 Centre Hospitalier, Ingbokolo Town, Democratic Republic of Congo; 7 Department of Infectiology & Hospital Hygiene, University Hospital Basel, Basel, Switzerland; Ministère de la Santé Publique et de la Lutte contre les Endémies, NIGER

## Abstract

**Background:**

Reducing morbidity is the main target of schistosomiasis control efforts, yet only rarely do control programmes assess morbidity linked to *Schistosoma* sp. infection. In the Democratic Republic of Congo (DRC), and particularly in north-eastern Ituri Province, little is known about morbidity associated with *Schistosoma mansoni* infection. For this reason, we aimed to assess intestinal and hepatosplenic morbidity associated with *S*. *mansoni* infection in Ituri Province.

**Methods/Principal findings:**

In 2017, we conducted a cross-sectional study in 13 villages in Ituri Province, DRC. *S*. *mansoni* infection was assessed with a Kato-Katz stool test (2 smears) and a point-of-care circulating cathodic antigen (POC-CCA) urine test. A questionnaire was used to obtain demographic data and information about experienced intestinal morbidity. Each participant underwent an abdominal ultrasonography examination to diagnose hepatosplenic morbidity. Of the 586 study participants, 76.6% tested positive for *S*. *mansoni*. Intestinal morbidity reported in the two preceding weeks was very frequent, and included abdominal pain (52.7%), diarrhoea (23.4%) and blood in the stool (21.5%). Hepatosplenic morbidity consisted of abnormal liver parenchyma patterns (42.8%), hepatomegaly (26.5%) and splenomegaly (25.3%). Liver pathology (adjusted odds ratio [aOR] 1.20, 95% confidence interval [CI] 1.06–1.37, *p* = 0.005) was positively and significantly associated with *S*. *mansoni* infection. Hepatomegaly (aOR 1.52, 95% CI 0.99–2.32, *p* = 0.053) and splenomegaly (aOR 1.12, 95% CI 0.73–1.72, *p* = 0.619) were positively but not significantly associated with *S*. *mansoni* infection at the individual level. At the village level, *S*. *mansoni* prevalence was positively associated with the prevalence of hepatomegaly and splenomegaly. High-intensity *S*. *mansoni* infections were associated with diarrhoea, blood in the stool, hepatomegaly, splenomegaly, and liver parenchyma (C, D, E and F pathology patterns). Four study participants were diagnosed with ascites and five reported hematemesis.

**Conclusions/Significance:**

Our study documents a high burden of intestinal and hepatosplenic morbidity associated with *S*. *mansoni* infection status in Ituri Province. The findings call for targeted interventions to address both *S*. *mansoni* infection and related morbidity.

## Author summary

Schistosomiasis caused by *Schistosoma mansoni* is of great public health importance in sub-Saharan Africa. The World Health Organization (WHO) recommends that control efforts aim to reduce morbidity through large-scale intervention programmes. However, intestinal and liver morbidity is rarely assessed in such control programmes. Hence, little is known about (i) the magnitude of the intestinal and liver morbidity burden in the community, or about (ii) the morbidity associated with *S*. *mansoni* infection, specifically. We conducted a cross-sectional study in which we assessed intestinal morbidity by questionnaire and liver morbidity by abdominal ultrasonography. Additionally, we determined the infection status of the study participants using standard diagnostic procedures (Kato-Katz technique and point-of-care cathodic circulating *S*. *mansoni* antigen [POC-CCA] urine test). Among the 586 study participants, aged six years and older, from 13 villages in Ituri Province, DRC, we observed a high degree of intestinal (23.4% with diarrhoea; 21.5% with blood in stool) and hepatosplenic morbidity (42.8% with abnormal liver C, D, E, and F patterns; 26.5% with an enlarged liver; 25.3% with an enlarged spleen). *S*. *mansoni* infection was associated with liver and spleen enlargement. Likewise, *S*. *mansoni* infection intensity was linked to diarrhoea, to liver and spleen enlargement and to pathological changes in the liver parenchyma. At village level, we observed that the prevalence of an enlarged liver and spleen among patients increased with the prevalence of *S*. *mansoni* infection. We conclude that the population of Ituri Province carries an alarming burden of intestinal, liver and spleen morbidity associated with *S*. *mansoni* infection. A comprehensive control programme to address this infection and disease burden is urgently required.

## Introduction

Schistosomiasis is a chronic helminth infection caused by trematodes of the genus *Schistosoma* and is one of the so-called neglected tropical diseases. It is a major cause of morbidity and mortality around the globe [1].

Depending on the species, the disease may be genitourinary (*Schistosoma haematobium*) or intestinal (*Schistosoma mansoni*, *S*. *japonicum*, *S*. *mekongi*, *S*. *intercalatum*, and *S*. *guineensis*). The disease arises from the host’s cell-mediated granulomatous immune response to the soluble antigens of the parasite eggs trapped in the tissues [2,3]. In the intestinal form of the disease, the adult worm dwells in the portal vein and mesenteric veinlets that drain the intestines, where the female deposits her eggs during her daily migration. Chronic and heavy infections are frequently associated with hepatosplenic and intestinal diseases, characterized by liver and spleen enlargements and intestinal damage. In the liver, the resulting scars may disrupt liver function and obstruct the portal veins, leading to periportal fibrosis (PPF), portal hypertension and, subsequently, to oesophageal varices, hematemesis, and melena and, ultimately, to ascites, the main cause of death due to *S*. *mansoni* infection [4,5]. In the intestines, inflammation may induce diarrhoea, while granulomas may cause polyposis with ulcers and recurrent bleeding. The resulting clinical manifestations may include abdominal pain, diarrhoea, and the presence of blood in the stool [2,6]. Chronic schistosomiasis can also lead to anaemia, stunted growth, and impairment of cognitive development [7,8]. Many infected people, even those with considerable infection intensity, may remain asymptomatic for a long period or experience only non-specific symptoms, such as nausea, headaches, fever, fatigue and abdominal pain [9].

Preventive chemotherapy (PC) through mass drug administration (MDA) is the WHO-recommended strategy for both reducing morbidity and controlling schistosomiasis in endemic settings [10]. However, PC is poorly implemented in many countries, often due to a lack of commitment or funding, and/or because of political instability and security issues, among other factors.

In the Democratic Republic of Congo (DRC), the full extent of the schistosomiasis morbidity burden remains unknown; relevant information is more than twenty years old [11,12]. Existing publications report on *Schistosoma* infection. The few reports related to morbidity mainly concern the province of Maniema, in the central-eastern region of the country [13,14]. In Ituri Province, morbidity due to *S*. *mansoni* infection was mentioned in colonial times [15]. Since then, only the colonial data and data from the 1970s and 1980s have been summarized in the available reviews. Madinga et al. [12] reported that *S*. *mansoni* endemic areas in Ituri were described before 1954, with prevalence rates ranging from 11.0% to 64.9% along the eastern bank of Lake Albert. Conversely, Gillet and Wolfs reported an absence of local cases in the high hill region, with prevalence ranging from 2.3% in Aru, in the north, to 93.7% in a fishing village on the shore of Lake Albert [15]. Neither review mentioned the existence of *S*. *haematobium* and *S*. *intercalatum* infections in Ituri [12,16]. While investigating the prevalence, intensity, and relative morbidity of *S*. *mansoni* infection among Ugandan and Zairian school children, aged 5 to 20 years, in Aru region, Müller et al. found that prevalence was low to moderately high. About 8.0% of children had heavy-intensity infections. Among the children, 15.6% to 38.0% had hepatomegaly, while 22.0% to 59.2% were diagnosed with splenomegaly. However, organomegaly associated with *S*. *mansoni* infection was insignificant [17].

To the best of our knowledge, the only study undertaken since the 1980s was the national survey conducted between 2013 and 2015. The survey results have not yet been published. However, several studies conducted in neighbouring Uganda show high *S*. *mansoni* infection and morbidity rates, and considerable mortality linked to infection [3,5,18]. The aim of the present study was to assess intestinal and hepatosplenic morbidity associated with *S*. *mansoni* infection in Ituri Province, DRC.

## Materials and methods

### Ethics statement

Patient examinations were part of a larger study, which was approved by the Swiss Ethical Commission (Ethikkommission in Nordwest- and Zentralschweiz, Ref. No. UBE-15/78) and by the University of Kisangani’s Research Ethical Commission, (Ref No: CER/003/GEAK/2016). Authorization was granted by the Nyankunde Higher Institute of Medical Techniques (Ref No 70/ISTM-N/SGAC/2017), Bunia, DRC. Permission for field work was obtained from the Ituri Provincial Health Division (Ref. 054/433/DPS/IT/06/2016 and Ref. 054/472/DPS/IT/06/2017) and from all relevant health districts. Prior to enrollment, the study objectives and procedures were explained in the local language to each participant and all of their questions were answered. Written informed consent was obtained from all study participants aged 15 years and older. Parents or legal guardians signed assent forms for participants aged 14 years and younger. Adolescents (15–17 years) signed the informed consent forms in agreement with the parents / guardians and in the presence of the village health volunteer. Participants diagnosed with S. mansoni were treated with praziquantel (40 mg/kg) [19]. All participants received Mebendazole (500 mg, single dose) for general deworming, in accordance with the DRC national deworming guidelines.

Written informed consent was obtained and included permission for taking photographs and publishing anonymized data.

### Study area

The study was conducted in Ituri Province, north-eastern DRC (geographical coordinates: 1.30°–3.60° latitude and 27.00°–31.40° longitude). Ituri has an area of 65,658 km^2^ and is home to 5.2 million inhabitants from five different ethnic groups (Nilo-Hamites, Bantu, Nilotic, Sudanese and Pygmy). The province is divided into five territories (counties) and 36 health districts. It is bordered by Lake Albert in the east, while several streams and rivers irrigate the province. These waterways are suitable environments for schistosomes’ snail intermediate host. For this study, six health districts were purposively selected because of the high prevalence of *S*. *mansoni* infection: Angumu, Bunia, Lolwa, Mandima, Nia-Nia and Tchomia. From these health districts, a total of 13 villages were purposively selected. From the biggest health district, Bunia, with a population of more than 500,000, six villages were selected (Lumumba, Simbilyabo, Kindia, Gupe, Sukisa, and Ngezi). Two villages were selected from Angumu (Gupe and Ndaru-Muswa), two from Lolwa (Mambau and Pekele), two from Nia-Nia (Bankoko and Mangenengene), one from Mandima (Mandima), and one from Tchomia (Kadjugi).

The presence of *S*. *mansoni* in the province was widely documented during colonial times, with transmission thought to occur mainly along the shores of Lake Albert [15]. Neither a review of the available literature nor a consultation with the provincial NTD control programme suggested the presence of *S*. *haematobium* in Ituri Province, nor did we find *S*. *haematobium* during our earlier work in the area [20]. For this reason, we concentrated our efforts on studying morbidity related to intestinal schistosomiasis caused by *S*. *mansoni* [12,15,16]. Only a small proportion of the population residing in Bunia has access to an adequate water supply. Most of the population uses natural water bodies (springs, ponds, and streams) as its main water source.

### Study design and population

We conducted a cross-sectional, household-based, in-depth study in 13 villages, purposively selected across six health districts in Ituri Province. Two-stage sampling procedures were used to select both households and individuals for the study. At least 10 households were randomly selected in each village, and all individuals aged six years and older and present on the day of the survey were enrolled. Household visitors, as well as mentally and terminally ill persons were excluded.

The study incorporated household and individual questionnaires; anthropometric assessments; and parasitological, clinical, and abdominal ultrasonographic examinations.

### Procedures

#### Individual questionnaires

All participants were invited to participate in an interview, conducted using a pre-tested questionnaire. The individual questionnaire focused on demographic, anthropometric, occupational, educational, and religious characteristics, as well as on knowledge, attitudes and practices related to *S*. *mansoni* infection and disease. The questionnaire also helped us to screen for signs and symptoms related to schistosomiasis, such as diarrhoea or blood in the stool in the two weeks preceding the study team visit, or a history of hematemesis at any time.

#### Anthropometric measurements

Participants’ weight and height were measured with a Seca analogue scale and height rod and reported to the nearest half kilogram (0.5 kg) and half centimetre (0.5 cm), respectively.

#### Parasitological examination

Participants were asked to provide one faecal sample (approx. 5 grams of morning stool) in a labelled plastic container for testing with the Kato-Katz technique [21]. From each stool specimen, two thick smears of 41.7 mg [21] were prepared and examined by experienced technicians. To allow for hookworm assessment, all smears were examined by microscope within one hour of preparation. All slides were examined for *S*. *mansoni* within 24 hours following stool collection. One third of the prepared smears were checked by the principal investigator. All helminth eggs were counted and recorded for each species separately. The intensity of the helminth infection was calculated by multiplying the mean number of eggs found on the two slides by 24. The result was expressed as eggs per gram (EPG) of stool [8].

Participants were also asked to provide a urine sample (approx. 60 ml) in a pre-labelled, wide-mouth, plastic container for the detection of circulating *S*. *mansoni* antigens using a point-of-care circulating cathodic antigen (POC-CCA) test. Both the stool and urine examinations were performed at the relevant village health centre facility.

The POC-CCA tests were performed according to the manufacturer’s guidelines (Rapid Medical Diagnostics, Pretoria, South Africa). Urine was examined on the day of collection. In cases where the test was postponed until the next day, urine samples were kept in a solar fridge, at 2–8°C (Steca, Germany). Test results were deemed negative if the POC-CCA band did not appear within 20 minutes. Trace, weak, medium, and strong coloured CCA bands were recorded as positive results. Questionable results were discussed among at least two technicians and the principal investigator.

#### Clinical examination

All participants underwent clinical and abdominal ultrasonography examinations. Clinical examinations consisted of physical checks performed by an experienced physician and assisted by an experienced nurse.

#### Abdominal ultrasound examination

An abdominal ultrasound was performed for each participant, in accordance with the Niamey protocol [22,23] and using a 2.0 MHz convex transducer U-Lite Sonoscanner Ultraportable HD Ultrasound Unit (U-Lite, Sonoscanner, 6, Rue André Voguet, Paris, France). A portable generator (MK, China) and solar powered batteries (for remote villages) were used as electricity sources.

The size of the left lobe, from the cranial to the caudal edge of the liver, was measured at about two centimetres from the xyphoid, in the left parasternal line (PSL). The length and width of the spleen were also measured, and its texture evaluated. All measures were taken in centimetres and performed using callipers, according to the manufacturer’s recommendations. Organ measurements were adjusted for the height of the individual and compared with those of healthy Cameroonian and Senegalese control groups. The left liver and spleen were considered enlarged if measurements exceeded the mean adjusted values for individuals in the control groups by two standard deviations (2 standard deviations [SD]) [24,25]. Liver parenchyma patterns (S1 Fig) were assessed following the WHO/TDR guidelines as follows: grade A, normal; grade B, incipient; grade C, probable; grades D, E and F, frank periportal fibrosis [22,23]. The diameter of the inner portal vein was measured. The length, width and wall thickness of the inner gall bladder were also measured. Other liver patterns that are not linked to schistosomiasis, such as fatty-liver-like (pattern Y) and other abnormalities (pattern Z) [22], were considered separately.

#### Data management and analysis

**Fig 1:**
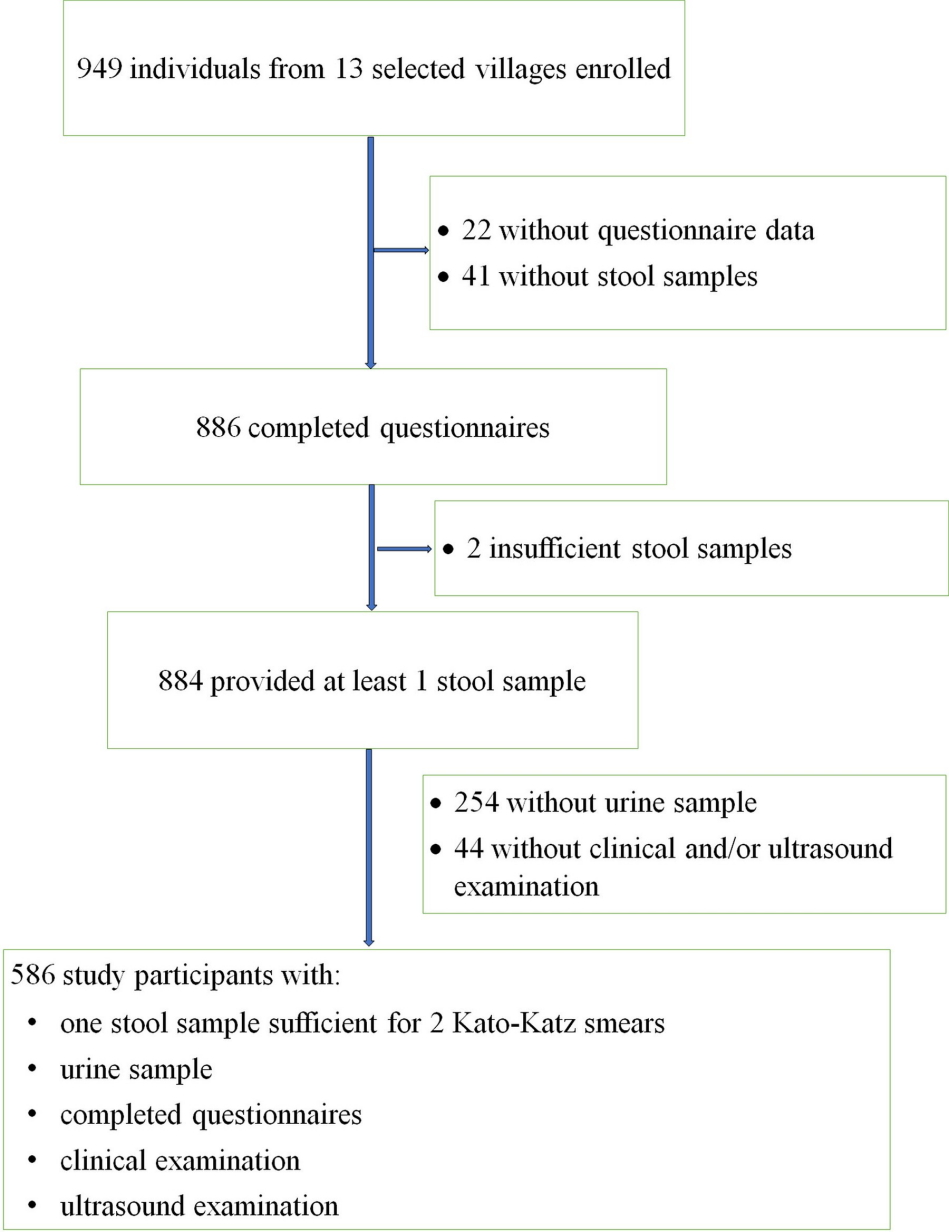
Flowchart of participant inclusion in the 2017 Ituri morbidity study across 13 villages.

Data was entered in Excel and cross-checked against the data sheet. STATA version 14.2 software (Stata Corp, College Station, USA) was used to manage and analyse data. Only participants with a complete dataset were retained in the analysis (Fig 1). Seven age groups were established: (i) 6–9 years, (ii) 10–14 years, (iii) 15–19 years, (iv) 20–29 years, (v) 30–39 years, (vi) 40–49 years and (vii) ≥50 years. Participants’ body mass index (BMI) was calculated (weight in kilograms divided by the square of the person’s height in metres, kg/m^2^), and four categories were set: underweight (<18.5 kg/m^2^), normal weight (18.5–24.9 kg/m^2^), overweight (25.0–29.9 kg/m^2^) and obese (≥30 kg/m^2^). Infection prevalence was expressed as the number of *S*. *mansoni-*positive individuals divided by the total number of participants examined. Infection intensity was estimated based on helminth egg counts per gram of stool (EPG) when examined with the Kato-Katz technique [21]. *S*. *mansoni* infection intensities were classified as light (1–99 EPG), moderate (100–399 EPG) and heavy (≥400 EPG) [8].

Arithmetic mean infection intensity was calculated. Categorical variables were presented as frequencies and percentages. Pearson’s chi-square (χ^2^) test was used to compare frequency distributions. A univariate logistic regression analysis was carried out to identify associations between *S*. *mansoni* infection status (outcome) and morbidity indicators (predictors) and/or demographic factors (age, gender). Predictors with a significance level of 20% or less, and age and gender variables were included in the multivariable logistic regression models. Odds ratio (OR), adjusted OR (aOR) and corresponding 95% confidence intervals (95% CI) were calculated. Only *p-*values <0.05 were considered statistically significant. In this study, we combined the results of the Kato-Katz technique and those of the POC-CCA test to diagnose infection status. For comparison, the same analysis was repeated for Kato-Katz and the POC-CCA diagnostic approaches, separately.

Ultrasonographic organ measurements were defined as enlarged if the left liver lobe and/or spleen length exceeded the normal reference value by 2 SD. Likewise, portal vein diameter was considered enlarged if it exceeded the normal reference value by 2 SD. Liver A and B patterns were considered normal, C and D patterns were deemed mild PPF, and E and F patterns were recorded as severe PPF. Liver Y and Z patterns were included in the analysis but neither as PPF nor normal patterns [22].

## Results

### Study population

Data were collected between June and September 2017. We enrolled participants from 13 purposively selected villages across six health districts with an anticipated high prevalence of *S*. *mansoni* infection. Of the 949 individuals enrolled (Fig 1), 586 (61.7%) completed all study procedures and had a complete dataset, that is, one stool sample examined with two Kato-Katz smears, a urine sample tested with POC-CCA, two completed questionnaires and a clinical and abdominal ultrasound examination.

Of those with a complete dataset, 342 (58.4%) were females, 330 (56.3%) were under 20 years of age and 268 (45.7%) were underweight (Table 1). The prevalence of *S*. *mansoni* was 59.2%, 65.7%, and 76.6% according to Kato-Katz, POC-CCA and combined test results, respectively. Thirty-seven percent, 15.2% and 7.2% of the population had light-, moderate- and heavy-intensity infections, respectively. Infection with soil transmitted helminths (STH) was not common among participants, with only eight participants diagnosed with an STH infection. In contrast, intestinal symptoms were very common, with 52.7%, 23.4% and 21.5%, reporting abdominal pain, diarrhoea, and blood in the stool within the two weeks preceding the survey, respectively. Five participants (0.9%) had experienced hematemesis at least once in his/her life. Abdominal ultrasound examinations revealed that 26.5% of participants had hepatomegaly, 25.3% had splenomegaly and 42.8% had liver pathology; 36.4% had mild PPF (C and D patterns), 6.4% had severe PPF (E and F patterns), 1.0% presented fatty liver (Y pattern), 0.2% had an unidentified abnormality (Z pattern) not linked to schistosomiasis and 0.7% had ascites. Only 56.0% of the participants had a normal and starry sky liver parenchyma (A and B patterns). More details on liver parenchyma patterns are shown in S7 and S8 Tables.

**Table 1:** Study population characteristics in the 2017 Ituri morbidity study. Study conducted in 13 purposively selected villages in Ituri Province (n = 586).

Characteristics		
	N	%
Gender		
Females	342	58.4
Males	244	41.6
Age categories (years)		
6–9	123	21.0
10–14	140	23.9
15–19	67	11.4
20–29	77	13.1
30–39	68	11.6
40–49	52	8.9
≥50	59	10.1
Body mass index (kg/m^2^—categories)		
Obese (≥30.0)	58	9.9
Overweight (25.0–29.9)	24	4.1
Normal weight (18.5–24.9)	236	40.3
Underweight (<18.5)	268	45.7
*S*. *mansoni* infection		
Kato-Katz test	347	59.2
CCA test	385	65.7
KK+CCA*	449	76.6
Infection intensity (KK only)		
Light	216	36.9
Moderate	89	15.2
Heavy	42	7.2
Soil transmitted helminths		
*Trichuris trichiura*	3	0.5
*Ascaris lumbricoides*	1	0.5
Hookworm	4	0.7
Clinical findings		
Diarrhoea	137	23.4
Blood in stool	126	21.5
Abdominal pain	309	52.7
Hematemesis	5	0.9
Ultrasound findings		
Hepatomegaly (US)	155	26.5
Splenomegaly (US)	148	25.3
Ascites	4	0.7
A and B patterns	328	56.0
C and D patterns	213	36.4
E and F patterns	38	6.4
Fatty liver	6	1.0
Other abnormality	1	0.2

* KK+CCA, combined any positive result by Kato-Katz and/or by point-of-care circulating cathodic antigen (POC-CCA); KK only, Kato-Katz results only with at least one egg in at least one of two smears.

### Morbidity associated with *S*. *mansoni* infection

**Fig 2:**
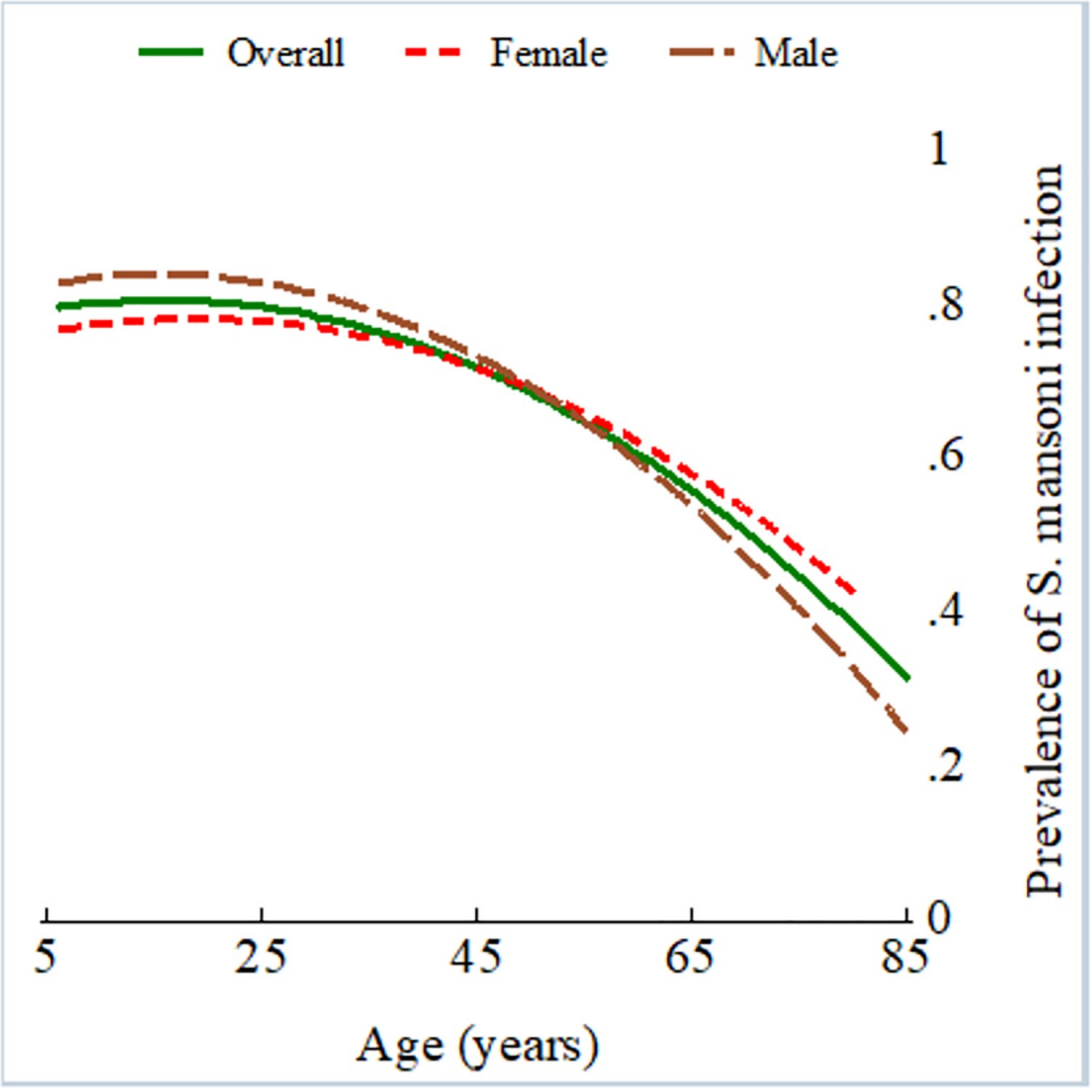
*S*. *mansoni* infection prevalence by age in the 2017 Ituri morbidity study (n = 586). Overall (green solid line), female (red dashed line), and male (maroon long-dashed line).

The results of the univariable risk analysis of the combined diagnostic approach are presented in Table 2. Male participants were more likely to be infected with *S*. *mansoni*, but the increased risk was not statistically significant (OR 1.22, 95% CI 0.82–1.81, *p* = 0.318). *S*. *mansoni* infection was observed more frequently in younger age groups, with prevalence peaking among young adults (Fig 2). Participants aged 50 years and older had a statistically significant reduced risk of infection compared to children aged 6–9 years (Table 2, OR 0.49, 95% CI 0.26–0.92, *P =* 0.024).

**Table 2:** Morbidity associated with *S*. *mansoni* infection in the 2017 study. Results of the univariable analysis of data from 13 purposively selected villages in Ituri Province (n = 586).

Characteristics	*S*. *mansoni* (+) N = 449 n		*S*. *mansoni* (-) N = 137 n		OR (95% CI)	*p*-value
Gender*						
Females	257	57.2	85	62.0	1.0	
Males	192	42.8	52	38.0	1.22 (0.82–1.81)	0.318
Age categories (years)*						
6–9	92	20.5	31	22.6	1.0	
10–14	119	26.5	21	15.3	1.61 (0.94–2.76)	0.078
15–19	54	12.0	13	9.5	1.10 (0.58–2.07)	0.769
20–29	63	14.0	14	10.2	1.53 (0.81–2.89)	0.184
30–39	48	10.7	20	14.6	0.77 (0.42–1.42)	0.396
40–49	35	7.8	17	12.4	0.73 (0.38–1.43)	0.359
≥50	38	8.5	21	15.3	0.49 (0.26–0.92)	0.769
STH						
*T*. *trichiura* (Y/N)	1	0.2	2	1.5	0.15 (0.01–1.69)	0.076
*A*. *lumbricoides* (Y/N)	1	0.2	0	0.0	na	
Hookworm (Y/N) *	4	0.2	3	2.2	0.10 (0.01–0.98)	0.015
Anthropometry (BMI)*						
Obese (Y/N)	36	8.0	22	16.1	1.0	
Overweight (Y/N)	18	4.0	6	4.4	1.83 (0.62–5.40)	0.264
Normal weight (Y/N)	183	40.8	53	38.7	2.11 (1.14–3.92)	0.016
Underweight (Y/N)	212	47.2	56	40.9	2.31 (1.25–4.28)	0.006
Clinical findings						
Diarrhoea (Y/N) *	114	25.4	23	16.8	1.69 (1.03–2.78)	0.038
Blood in stool (Y/N)	99	22.1	27	19.7	1.15 (0.72–1.86)	0.560
Abdominal pain (Y/N)	238	53.0	71	51.8	1.05 (0.72–1.54)	0.808
Hematemesis (Y/N)	4	0.9	1	0.7	1.22 (0.14–11.05)	0.858
Ultrasound findings						
Hepatomegaly (Y/N) *	125	27.8	30	21.9	1.38 (0.87–2.17)	0.168
Splenomegaly (Y/N) *	118	26.3	30	21.9	1.27 (0.81–2.01)	0.302
Ascites (Y/N) *	2	0.5	2	1.5	0.30 (0.04–2.17)	0.207
A/B patterns (Y/N) *	246	54.8	82	59.9	1.0	
C/D patterns (Y/N)	167	37.2	46	33.6	1.21 (0.80–1.83)	0.363
E/F patterns (Y/N)	30	6.7	8	5.8	1.25 (0.55–2.84)	0.593
Fatty liver (Y/N)	5	1.1	1	0.7	2.00 (0.24–16.94)	0.696
Other (Y/N)	1	0.2	0	0	na	0.580

* Included in the multivariable analysis. BMI, body mass index; na, not applicable; A pattern: normal; B pattern: “starry sky”; C pattern: “rings and pipe-stems”; D pattern: “highly echogenic ruff around portal bifurcation”; E pattern: “highly echogenic patches”; F pattern: “highly echogenic bands and streaks–bird’s claw”; Fatty liver (Y pattern) and other abnormality (Z pattern) indicate pathology different from periportal fibrosis [22,23].

Intestinal helminth coinfections were negatively associated with *S*. *mansoni* infection status; of these, the association with hookworm infection was statistically significant (OR 0.10, 95% CI 0.01–0.98, *p* = 0.015). Participants who reported an episode of diarrhoea within the two weeks preceding the study had an increased risk of *S*. *mansoni* infection (OR 1.69, 95% CI 1.03–2.78, *p* = 0.038).

Diagnosed hepatomegaly (OR 1.38, 95% CI 0.87–2.17, *p* = 0.168), splenomegaly (OR 1.27, 95% CI 0.81–2.01, *p* = 0.302) and E/F liver parenchyma patterns (OR 1.25, 95% CI 0.55–2.84, *p* = 0.593) were positively but not significantly associated with an *S*. *mansoni* infection.

The univariable risk analyses of the Kato-Katz and POC-CCA diagnostic approaches are given in supplementary S1 and S3 Tables, respectively, and show a very similar risk pattern. It is worth noting that when the results of the Kato-Katz tests were considered alone, male participants had a significantly higher risk of *S*. *mansoni* infection (S1 Table, OR 1.44, 95% CI 1.03–2.03, *p* = 0.033); and, unlike splenomegaly (S1 Table, OR 1.61, 95% CI 1.09–2.39, *p* = 0.017), hepatomegaly (S1 Table, OR 1.41, 95% CI 0.96–2.06, *p* = 0.079) was not significantly associated with *S*. *mansoni* infection.

**Fig 3:**
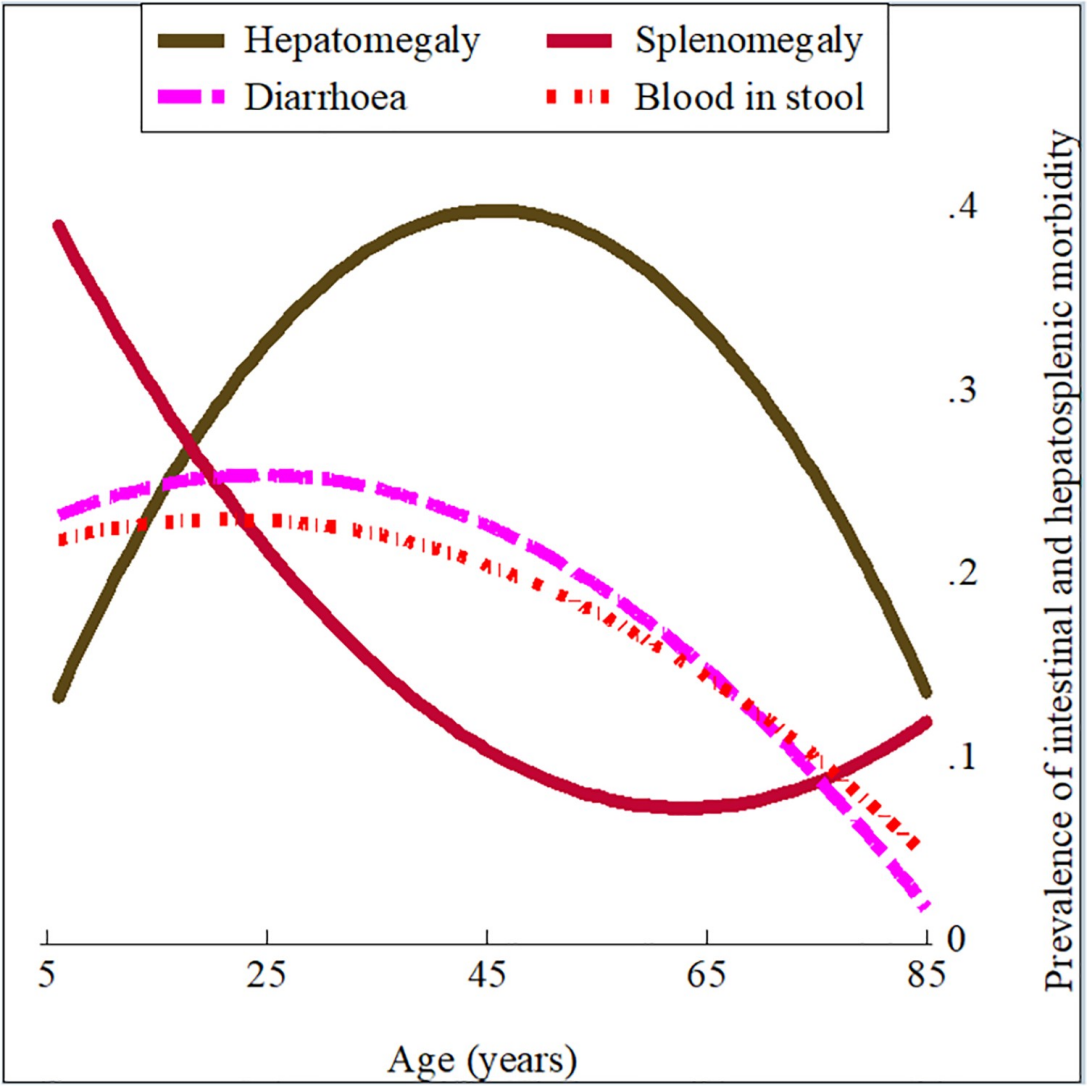
Age distribution of intestinal and hepatosplenic morbidity in the 2017 Ituri morbidity study (n = 586). Hepatomegaly (olive solid line), splenomegaly (cranberry solid line), diarrhoea (magenta long-dashed line), and blood in stool (red short-dash-dot line).

Reported diarrhoea and blood in the stool, as well as the ultrasonographically assessed hepato- and splenomegaly displayed an age distribution resembling that of *S*. *mansoni* infection levels, with corresponding peaks in the adolescent and adult age groups (Fig 3).

The risk analysis took into account the results of the combined diagnostic approach (Table 3). The results were consistent with those of the single diagnostic approaches, using Kato-Katz or POC-CCA only. However, there were some differences. First, intestinal morbidity indicators, such as diarrhoea, were significantly associated with an *S*. *mansoni* infection (OR 1.69, 95% CI 1.03–2.78, *p* = 0.038). This was also the case when the results of the Kato-Katz diagnostic approach were analysed (OR 1.85, 95% CI 1.22–2.79, *p* = 0.003). However, the association was not significant (OR 1.42, 95% CI 0.93–2.16, *p* = 0.101) for the results of the POC-CCA diagnostic approach. Other indicators, including the presence of blood in the stool, abdominal pain, and history of hematemesis, showed no association, regardless of the diagnostic approach used. Second, hepatomegaly was not significantly associated with an *S*. *mansoni* infection when analysing the results of the combined diagnostic approach (OR 1.38, 95% CI 0.87–2.17, *p* = 0.168), the POC-CCA diagnostic approach (OR 1.33, 95% CI 0.89–1.98, *p* = 0.158), or the Kato-Katz diagnostic approach (OR 1.41, 95% CI 0.96–2.06, *p* = 0.079). Splenomegaly was significantly associated with an *S*. *mansoni* infection when analysing the Kato-Katz diagnostic results (OR 1.61, 95% CI 1.09–2.39, *p* = 0.017). The association was not statistically significant for the combined (OR 1.27, 95% CI 0.81–2.01, *p* = 0.302) or the POC-CCA (OR 1.17, 95% CI 0.78–1.74, *p* = 0.451) diagnostic approaches. Third, an abnormal liver parenchyma pathology (combined E/F patterns) was significantly associated with *S*. *mansoni* infection when analysing the Kato-Katz diagnostic results (OR 2.25, 95% CI 1.05–4.80, *p =* 0.032). The association was not significant for POC-CCA (OR 1.20, 95% CI 0.58–2.47, *p* = 0.618) or the combined approach (OR 1.25, 95% CI 0.55–2.84, *p* = 0.593).

**Table 3:** Morbidity associated with *S*. *mansoni* infection in the 2017 study based on Kato-Katz and POC-CCA diagnostic approaches. Results of the multivariable analysis of data from 13 purposively selected villages in Ituri Province (n = 586).

Risk factors	aOR (95% CI)	Std. Err.	z	*p*-value
Demographic risk factors				
Age	0.98 (0.96–0.99)	0.006	-3.64	<0.001
Gender (Male/Female)	1.15 (0.74–1.79)	0.259	0.64	0.524
Anthropometric risk factors				
BMI	1.00 (0.95–1.06)	0.027	0.15	0.878
Clinical finding				
Diarrhoea	1.69 (0.99–2.89)	0.461	1.94	0.053
Blood in stool	0.90 (0.54–1.50)	0.237	-0.41	0.683
Ultrasound findings				
Hepatomegaly (Yes/No)	1.58 (0.96–2.61)	0.404	1.80	0.071
Splenomegaly (Yes/No)	0.91 (0.55–1.50)	0.230	-0.37	0.712
Ascites (Yes/No)	0.21 (0.03–1.69)	0.225	-1.46	0.144
Liver pathology (Yes/No)	1.13 (0.98–1.31)	0.085	1.65	0.100
Coinfection				
Hookworm (Yes/No)	0.08 (0.01–0.81)	0.093	-2.14	0.033

aOR: adjusted odds ratio in multivariable analysis; CI: confidence interval; BMI: body mass index (continuous variable).

Ten variables were included in the multivariable logistic regression analysis, the results of which are displayed in Table 3. Age was negatively associated with *S*. *mansoni* infection (adjusted odds ratio [aOR] 0.98; 95% CI 0.96–0.99, *p* = <0.001), while gender was not significantly associated with *S*. *mansoni* infection (aOR 1.15; 95% CI 0.74–1.79, *p* = 0.524).

Of the morbidity indicators investigated, diarrhoea (aOR 1.69; 95% CI 0.99–2.89, *p* = 0.053) and hepatomegaly (aOR 1.58; 95% CI 0.96–2.61, *p* = 0.071) were associated with *S*. *mansoni* infection with a borderline significance level. Patients with abnormal liver parenchyma patterns (aOR 1.13; 95% CI 0.98–1.31, *p* = 0.100) did not have an increased risk for *S*. *mansoni* infection.

**Fig 4:**
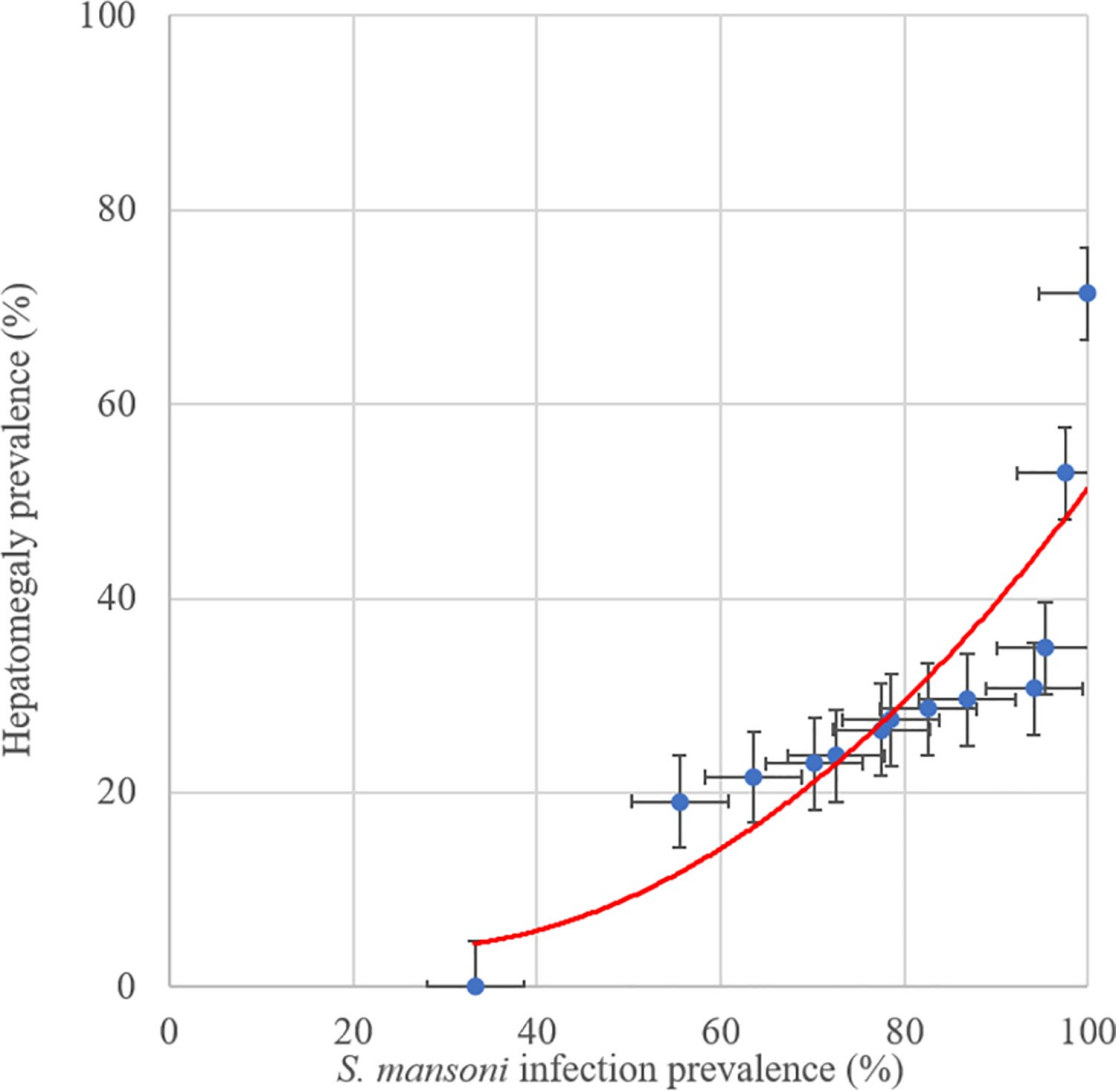
Association of hepatomegaly and *S*. *mansoni* infection prevalence at village level in the 2017 Ituri morbidity study (n = 586).

**Fig 5:**
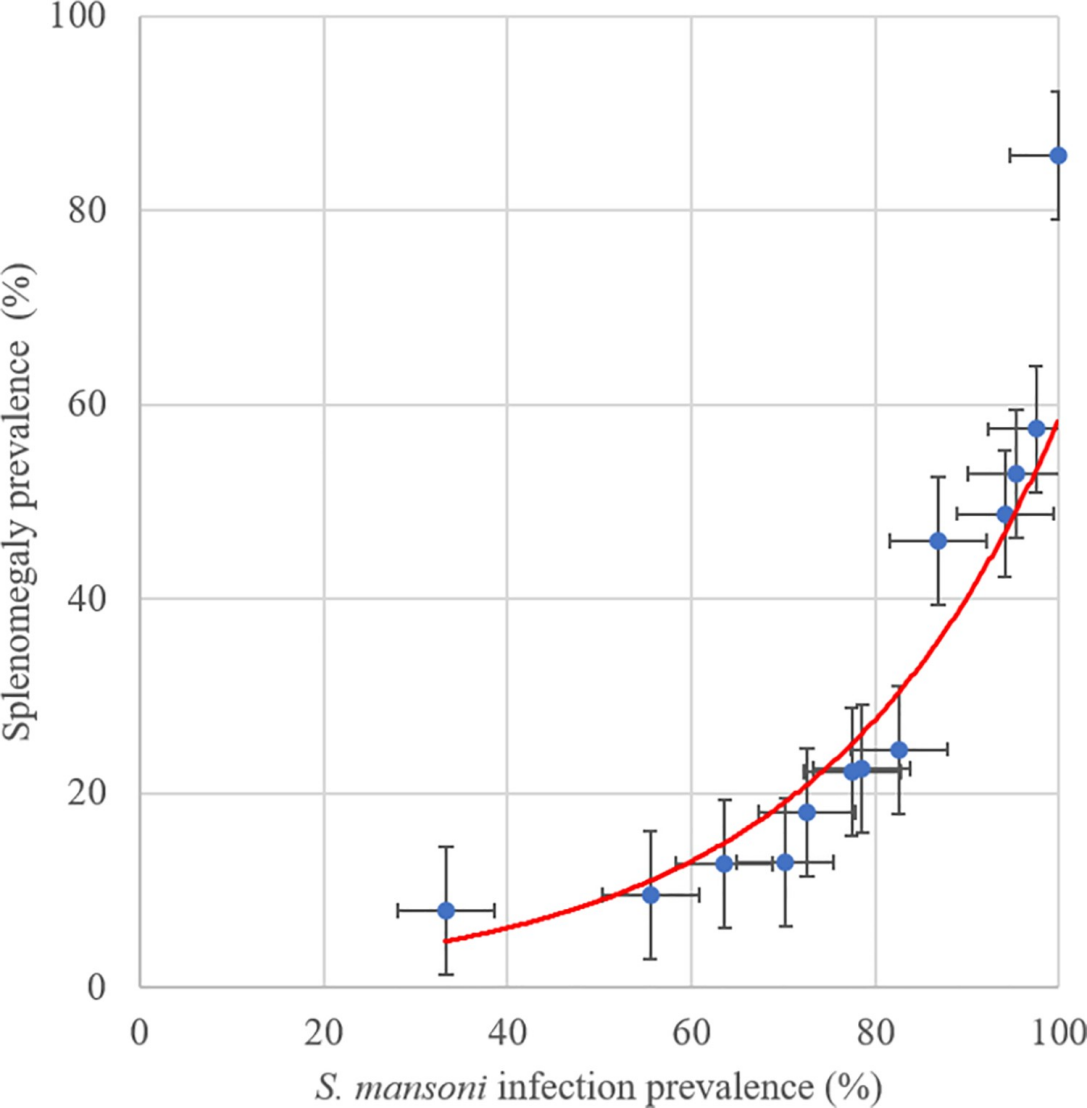
Association of splenomegaly and *S*. *mansoni* infection prevalence at village level in the 2017 Ituri morbidity study (n = 586).

At the village level, the prevelance of hepatomegaly (Fig 4) and splenomegaly (Fig 5) increased with the prevalence of *S*. *mansoni* infection. Four patients were diagnosed with ascites all of whom were residents of villages where overall *S*. *mansoni* prevalence exceeded 80%.

*S*. *mansoni* infection intensity varied greatly among individuals, with a maximum of 4,497.6 EPG and a mean infection intenstity of 109.7 EPG. Table 4 presents the infection intensity according to gender, age, helminth coinfection and morbidity categories. Infection intensity levels were similar in the two gender groups (*p* = 0.198). The age distribution of the infection intensity levels followed the age-infection prevalence curve. Heavy-intensity infections were mostly found (12.9%) among adolescents, aged 10–14 years, while no one in the oldest age group (50 years and older) had a heavy-intensity infection (*p* = 0006). Heavy-intensity infections were significantly higher among patients co-infected with *Ascaris lumbricoides* (13.0%, *p* = 0.005) and underweight participants (10.1%, *p* = 0.033).

**Table 4:** *S*. *mansoni* infection intensity by morbidity in the 2017 study. Study conducted in 13 purposively selected villages in Ituri Province (n = 586). Only results of the Kato-Katz diagnostic approach have been considered in this analysis.

Characteristics			*S*. *mansoni* infection intensity							
	Negative		Light		Moderate		Heavy		χ^2^	*p*-value
	n	%	n	%	n	%	n	%		
Overall	239	40.8	216	36.9	89	15.2	42	7.2		
Gender										
Females	152	44.4	117	34.2	50	14.6	23	6.7		
Males	87	35.7	99	40.6	39	16.0	19	7.8	4.66	0.198
Age categories (years)										
6–9	50	40.7	47	38.2	18	14.6	8	6.5		
10–14	49	35.0	49	35.0	24	17.1	18	12.9		
15–19	19	28.0	25	37.3	17	25.4	6	9.0		
20–29	27	35.0	32	41.6	11	14.3	7	9.1		
30–39	31	45.6	26	38.2	9	13.3	2	2.9		
40–49	27	51.9	18	34.6	6	11.6	1	1.9		
≥50	36	61.0	19	32.2	4	6.8	0	0.0	36.68	0.006
Soil-transmitted helminths										
*T*. *trichiura* (Y/N)	2	0.8	1	0.5	0	0.0	0	0.0	1.18	0.758
*Ascaris* (Y/N)	0	0.0	0	0.0	0	0.0	1	2.4	13.0	0.005
Hookworm (Y/N)	3	1.3	1	0.5	0	0.0	0	0.0	2.21	0.530
Anthropometry (BMI)										
Obese	33	56.9	20	34.5	4	6.9	1	1.7		
Overweight	13	54.2	9	37.5	2	8.3	0	0.0		
Normal weight	96	40.7	89	37.7	37	15.7	14	5.9		
Underweight	97	36.2	98	36.5	46	17.2	27	10.1	18.15	0.033
Clinical findings										
Diarrhoea (Y/N)	41	17.2	56	25.9	23	25.8	17	40.5	13.11	0.004
Blood in stool (Y/N)	42	17.6	32	14.8	30	33.7	22	52.4	39.49	<0.001
Abdom. pain (Y/N)	124	51.9	107	49.5	52	58.4	26	61.9	3.53	0.317
Hematemesis (Y/N)	3	1.3	1	0.5	1	1.1	0	0.0	1.28	0.733
Ultrasound findings										
Hepatomegaly (Y/N)	54	22.6	65	30.1	20	22.5	16	38.1	6.95	0.073
Splenomegaly (Y/N)	48	20.1	48	22.2	28	31.5	24	57.1	28.88	<0.001
Ascites (Y/N)	3	1.3	0	0.0	1	1.1	0	0.0	3.19	0.364
A/B patterns (Y/N)	146	44.5	120	36.6	43	13.1	19	5.8		
C/D patterns (Y/N)	80	37.6	79	37.1	37	17.4	17	8.0		
E/F patterns (Y/N)	10	26.3	13	34.2	9	23.7	6	15.8		
Fatty liver (Y/N)	2	33.3	4	66.7	0	0.0	0	0.0		
Other (Y/N)	1	100	0	0.0	0	0.0	0	0.0		

BMI: body mass index; Abdom.: abdominal. A pattern: normal; B pattern: “starry sky”; C pattern: “rings and pipe-stems”; D pattern: “highly echogenic ruff around portal bifurcation”; E pattern: “highly echogenic patches”; F pattern: “highly echogenic bands and streaks–bird’s claw”; Fatty liver and Other non-identified pathology indicate pathology different from periportal fibrosis. [22,23].

There was a significantly higher prevalence of reported diarrhoea (40.5%, *p* = 0.004) and blood in the stool (52.4%, *p*<0.001) among patients in the heavy-intensity infection group compared to the other infection intensity groups.

**Fig 6:**
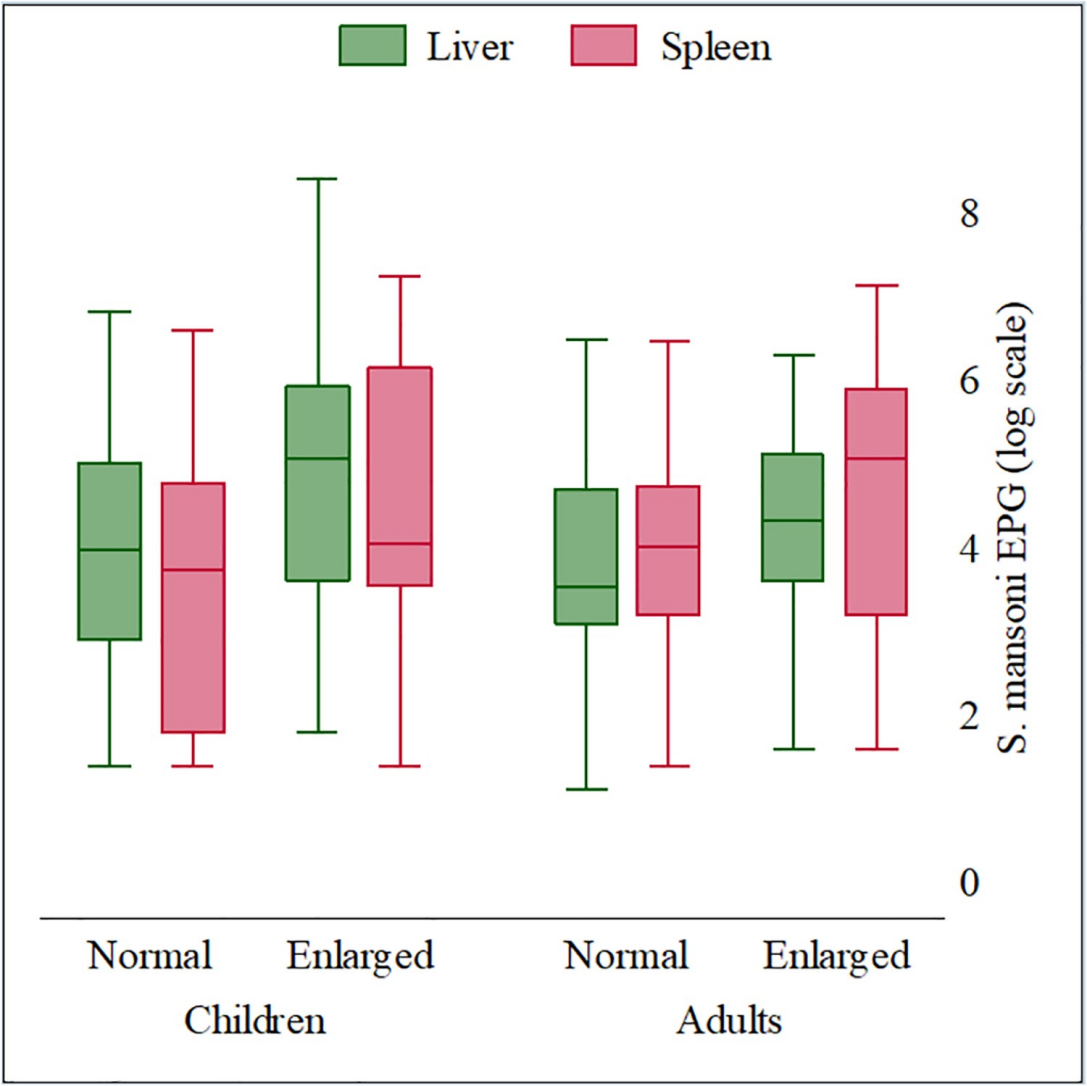
*S*. *mansoni* infection intensity by hepatomegaly and splenomegaly and age in the 2017 Ituri morbidity study (n = 586). Hepatomegaly (green) and splenomegaly (cranberry).

Among patients with heavy-intensity infections, the prevalence of splenomegaly (57.1%) was significantly higher than among other infection intensity groups (*p*<0.001), while the prevalence of hepatomegaly (38.1%) was not statistically different from other infection intensity groups (*p* = 0.073). When stratified by age, patients with an enlarged liver and/or spleen bore a higher infection intensity burden compared to those with a normal-sized liver and spleen in the same age group (Fig 6 and S6 Table). In general, younger patients (children <18 years old) experienced more high-intensity infections compared to those in the older age groups (adults ≥ 18 years old).

**Fig 7:**
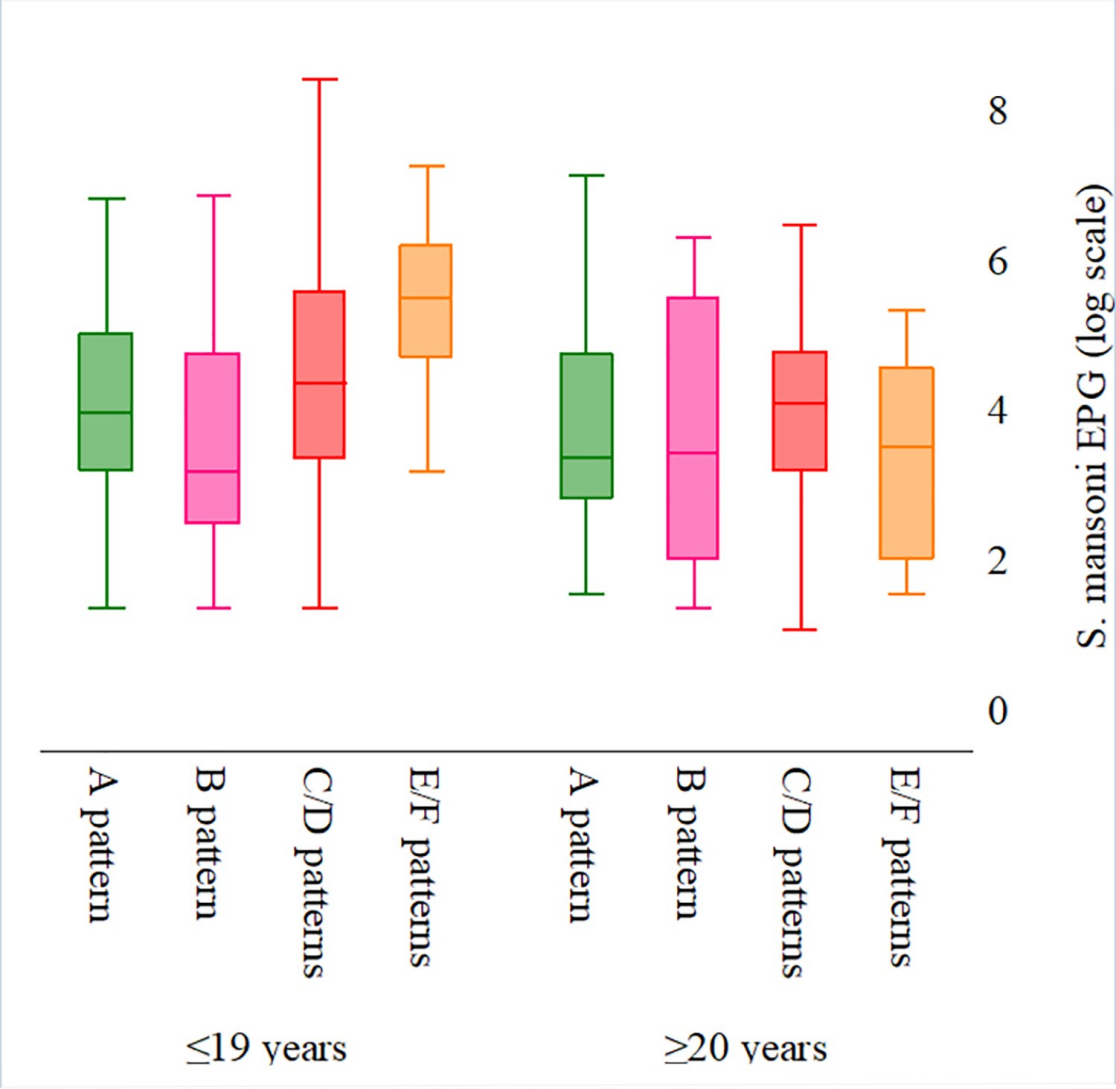
*S*. *mansoni* infection intensity by liver parenchyma patterns and age in the 2017 Ituri morbidity study (n = 586). A (dark green) and B (purple) patterns: normal; C (maroon) and D (cranberry) patterns: mild PPF; E (red) and F (orange) patterns: severe PPF; Fatty liver (pink) [22].

*S*. *mansoni* infection intensity varied considerably among patients with different liver parenchyma pathologies (S5 and S7 Tables). In general, more heavy-intensity infections were observed among patients with more severe liver morbidity patterns. That is, the number of individuals with heavy-intensity infections increased with the severity of the liver parenchyma pattern, from normal and starry sky (A and B) liver parenchyma patterns (5.8%), to the most severe (“bird’s claw”) E and F patterns (15.8%). The association was not statistically significant (*p* = 0.107). When stratified by age, a clear association emerged between increased number of high-intensity infections and increasingly abnormal liver pathologies (Fig 7). Liver parenchyma worsened (from normal/starry sky (A and B) patterns, to mild PPF C and D patterns, and to severe PPF E and F patterns) as the median infection intensity increased. However, taken alone, patients with F pattern had similar or lower-intensity infections than patients with less severe morbidity patterns (S7 Table).

## Discussion

In the World Health Organization’s roadmap for neglected tropical diseases 2012–2030, the focus with regard to schistosomiasis is morbidity control. This is especially the case for the African region, where transmission levels in several countries, including the DRC, are high, and elimination is not yet on the horizon. A key recommendation is to administer preventive chemotherapy, namely praziquantel. Mass treatment of a risk-exposed population at regular intervals prevents the development of high-intensity infections and hence, of morbidity [26,27].

Our study provides, for the first time, comprehensive baseline data showing a high intestinal and hepatosplenic morbidity burden associated with *S*. *mansoni* infection in Ituri Province, at both the individual and community levels.

Although minimizing morbidity is the target of schistosomiasis control efforts, control programmes rarely collect (baseline) and monitor morbidity data. Instead, they largely rely on monitoring infection intensities, which are linked to morbidity and much easier to assess than intestinal and hepatosplenic morbidity. Consequently, little is known about the morbidity burden of schistosomiasis [6].

Control programmes were conducted successfully in colonial times, even with means that today may be considered outdated [15]. Following independence, no large-scale activity aimed at combating the disease had been undertaken until 2012. A national public control programme targeting school children started in the country and was launched in Ituri Province in 2016.

We found a high degree of intestinal and hepatosplenic morbidity. About one quarter of the study participants reported diarrhoea and blood in the stool. Upon ultrasonography examination, almost one-quarter was diagnosed with hepatomegaly; almost two-thirds had splenomegaly, and more than half had an abnormal liver parenchyma (C–F patterns). Five patients reported an experience of hematemesis and four patients had ascites.

The prevalence of *S*. *mansoni* infection was high among the study population. As the POC-CCA diagnostic approach does not provide information about infection intensity, the Kato-Katz technique was used to analyse infection intensity. Light-, moderate- and heavy-intensity infections were diagnosed in high frequencies. Only a few cases of soil-transmitted helminths were diagnosed during this study and likely had a very small impact on the morbidity findings. *S*. *mansoni* infection prevalence and intensity was highest in the adolescent and young adult age groups. The prevalence of intestinal and hepatosplenic morbidity indicators showed a very similar age distribution (although hepato- and splenomegaly peaked in older age groups); at village level, hepatosplenic morbidity prevalence increased with infection prevalence. Both observations suggest a close link between morbidity and *S*. *mansoni* infection. Furthermore, at individual level, we found an increased risk of hepatomegaly and splenomegaly in *S*. *mansoni-*infected patients, consistent with the association found at village level and the aforementioned age distribution. The findings are also consistent with documented hepatosplenic morbidity associated with *S*. *mansoni* infection [28,29], hence, providing further evidence that *S*. *mansoni* infection is a major contributor to the overall observed morbidity.

We found three notable differences in risk when relying on the diagnostic results from the Kato-Katz technique only. However, as we sought the respective advantages of a more specific (Kato-Katz) and a more sensitive (POC-CCA) diagnostic approach, we considered the results of the combined diagnostic approach. First, reported diarrhoea was significantly associated with *S*. *mansoni* infection; second, pathological changes of the liver parenchyma were associated with *S*. *mansoni* infection as was hepatomegaly; and third, splenomegaly was not associated with *S*. *mansoni* infection. Using the Kato-Katz test alone to diagnose *S*. *mansoni* infection reduces the overall sensitivity of the diagnostic approach due to the low sensitivity of the technique itself [30,31]. Hence, on average, those diagnosed with an *S*. *mansoni* infection via the KK test are more likely to have a higher-intensity infection compared to those diagnosed with the combined approach. From this observation, we see that subtle increases in morbidity—such as reported diarrhoea and pathological changes in the liver parenchyma—become statistically significant. Indeed, for both morbidity indicators, we observed an association with *S*. *mansoni* infection intensity. Patients with diarrhoea had the highest prevalence of heavy-intensity infections (Table 2) and those with abnormal liver parenchyma E–F patterns had the highest mean infection intensities (Fig 4 and S7 Table). However, the association between parasitological diagnosis and morbidity was much higher at the community (village) level than at the individual level.

We found that patients with abnormal liver parenchyma pattern F displayed lower *S*. *mansoni* infection intensity and risk compared to those with E pattern. The highly echogenic bands and streaks corresponding to F pattern extend from the main portal vein and its bifurcation to the liver surface. Most commonly, this pattern manifests itself in very advanced cases of liver fibrosis and is frequently accompanied by other changes [22,23]. Patients who displayed pattern F were often older, and thus had little contact with water, while some had already been treated multiple times with praziquantel. Consequently, they may have been free of infection or may have had light-intensity infections. It is known that treatment with praziquantel cannot halt the progression of organ damage in some individuals. This situation may be due to immunological and genetic factors, as well as other influences, including malaria, viral hepatitis and/or concomitant alcohol consumption [5,32–34].

Quantifying schistosomiasis morbidity is a challenging [6,35] and controversial matter [4]. Morbidity associated with *Schistosoma* infection is unspecific. Hence, the morbidity pattern observed might have been provoked partially by or in combination with other pathogens, such as other helminth species, protozoa, bacteria, and viruses. Given that multiple infections are frequent in tropical Africa, a combination of infections is most likely responsible for the observed morbidity. In Ituri Province, other parasitic infections, such as malaria (*Plasmodium falciparum*), and other infections with hepatosplenic affinity, such as viral hepatitis, are prevalent [3,5,36–37] and may have contributed to the hepatosplenic morbidity pattern observed. The time gap between infection and the occurrence of measurable morbidity further complicates efforts to assess the association between infection and morbidity. Furthermore, organomegaly is sometimes described as normally present in children; it then regresses and disappears in adulthood [5,38–40].

Nevertheless, these findings do not in any way reduce the value of abdominal ultrasound in diagnosing liver pathologies associated with *S*. *mansoni* infection. However, ultrasonography devices are rarely available in poor-resource settings. In Ituri, the device may be found only in the provincial hospital, and in some district hospitals and private clinics. During our field work, we found that the use of portable devices was feasible in the villages. However, in the most remote rural areas, usage is challenging due to the unavailability of electricity. Additional equipment, such as solar panels and rechargeable batteries, are required. Despite these shortcomings, abdominal ultrasonography yields crucial information about liver morbidity in the community and hence, indispensable information about the public health burden of *S*. *mansoni* infection [41]. Severe cases are likely to be diagnosed earlier and adequately managed.

We encountered patients with severe complications from *S*. *mansoni* infection, which further underscored the importance of the infection’s morbidity burden. Four people (0.9%) reported a history of hematemesis and two people (0.5%) reported ascites. The finding appears to corroborate the health service’s statistics report from the Angumu health district (on the shore of Lake Albert), which declares that hematemesis is a frequent medical emergency and that adults in this area have died after vomiting blood. Oesophageal varices remain silent until they rupture and irreversible damage occurs [37]. Angumu health district is a remote area and well known for its high blood transfusion rates. Patients vomiting blood often reach the hospital too late, leading to the worst medical outcome.

The morbidity levels we observed are consistent with those measured by Ongom and Bradley [18], who found serious morbidity, including diarrhoea and abdominal pain, in a schistosomiasis endemic community on Lake Albert in Uganda. Other studies of *S*. *mansoni* endemic communities outside of the DRC present similar morbidity levels [40–42]. Very few studies of morbidity due to schistosome infection in DRC exist [37,43]. Our study contributes to the country’s knowledge base and may offer a baseline for future intervention studies to determine the exact extent of morbidity associated with *S*. *mansoni* infection.

The study presents some limitations. As the study was conducted in purposively sampled villages known for their high prevalence of *S*. *mansoni* infection, the examined population is not representative of the entire province, but rather of high transmission areas. Furthermore, ongoing civil unrest in Ituri creates a challenging security situation, which only afforded us a short time in each village. For this reason, only one stool sample could be collected from each study participant. Finally, limited available resources did not allow us to examine participants for parasitic, bacterial, and viral coinfections, which could have helped to better explain the degree of morbidity linked to *S*. *mansoni* infection.

Other limitations include a lack of blood coinfection diagnosis and a body mass index (BMI) cut-off that did not take into account variations among the study population, as recommended [44]. Concerning the first, we needed to minimize invasive procedures and exclude vulnerable individuals. Indeed, blood sampling is a very sensitive topic in our study area, as the population is reluctant to have blood drawn. Blood sampling would have required a lot of time and effort and would have greatly reduced the level of compliance among the study participants. Hence, we did not diagnose coinfections such as malaria [45], viral hepatitis [46], human immunodeficiency virus (HIV), and other infections endemic in the province. Therefore, alcoholism or other infections and afflictions prevailing in Ituri Province [47] might have contributed to the observed morbidity, thereby downplaying the role of *S*. *mansoni* infection in the hepato-splenic pathology.

The BMI cut-off values were defined by four categories, including underweight (<18.5 kg/m^2^), normal weight (18.5–24.9 kg/m^2^), overweight (25.0–29.9 kg/m^2^) and obese (≥30 kg/m^2^). This clustering may have introduced a selection bias in our results. However, in the risk analysis, we used the BMI as a continuous variable rather than a categorical one.

## Conclusion

*Schistosomiasis mansoni* is an important public health problem in Ituri Province, yet appropriate control measures have not yet been fully implemented. A public programme for controlling the disease was launched in the province in 2016. It is based on preventive chemotherapy and aims to control infection among school children. Our results show that both infection rates and related morbidity are very high in the province. However, other unexplored factors could have contributed to the morbidity observed. The situation calls for vigorous and efficient control measures to address this scourge.

## Supporting information

S1 Fig Liver image patterns associated with schistosomiasis, by [23]. (TIF)

S1 Table Univariable associations with *S*. *mansoni* infection in the 2017 morbidity study. Study conducted in 13 purposively selected villages in Ituri province (n = 586). Only diagnostic results of Kato-Katz (KK) tests have been considered.
(DOCX)

S2 Table Risk factors for morbidity due to *Schistosoma mansoni* infection, 2017 study. Results of the multivariable analysis of risk factors for morbidity due to *Schistosoma mansoni* infection among participants from 13 villages in Ituri province (n = 586). Only diagnostic results of Kato-Katz (KK) tests have been considered.
(DOCX)

S3 Table Univariable associations with *S*. *mansoni* infection in the 2017 morbidity study. Study conducted in 13 purposively selected villages in Ituri province (n = 586). Only diagnostic results of the point-of-care circulating cathodic antigen (POC-CCA) tests have been considered.
(DOCX)

S4 Table Risk factors for morbidity due to *Schistosoma mansoni* infection, 2017 study. Results of the multivariable analysis of risk factors for morbidity due to *Schistosoma mansoni* infection among participants from 13 villages in Ituri province (n = 586). Only results of the point-of-care circulating cathodic antigen (POC-CCA) diagnostic tests have been considered.
(DOCX)

S5 Table Prevalence of periportal fibrosis (PPF) by age, sex, village, and *S*. *mansoni* infection status in the 2017 study. Results from 13 purposively selected villages of Ituri province (n = 586). Prevalence derived from combined diagnostic approach and intensity determined by Kato-Katz test results.
(DOCX)

S6 Table Prevalence of hepatomegaly, splenomegaly, and overall organomegaly by age, sex, village, and *S*. *mansoni* infection status in the 2017 study. Results from 13 purposively selected villages in Ituri province (n = 586). Prevalence derived from a combined diagnostic approach.
(DOCX)

S7 Table *S*. *mansoni* infection intensity by liver patterns in the 2017 study. Study conducted in 13 purposively selected villages in Ituri province (n = 586). Only results of the Kato-Katz diagnostic tests have been considered in this analysis.
(DOCX)

S8 Table Univariable analysis of risk of liver patterns and periportal fibrosis (PPF) by *S*. *mansoni* infection status with different diagnostic approaches in the 2017 study. Results from 13 purposively selected villages of Ituri province (n = 586). Analysis of Kato-Katz results, POC-CCA results, and the combined diagnostic approach results.
(DOCX)
