# Supplementary figures and images for: Morbidity associated with Schistosoma mansoni infection in north-eastern Democratic Republic of the Congo
Source: PLoS Negl Trop Dis. 2021 Dec 2;15(12):e0009375. doi: 10.1371/journal.pntd.0009375 (PMC8638987; doi:10.1371/journal.pntd.0009375)

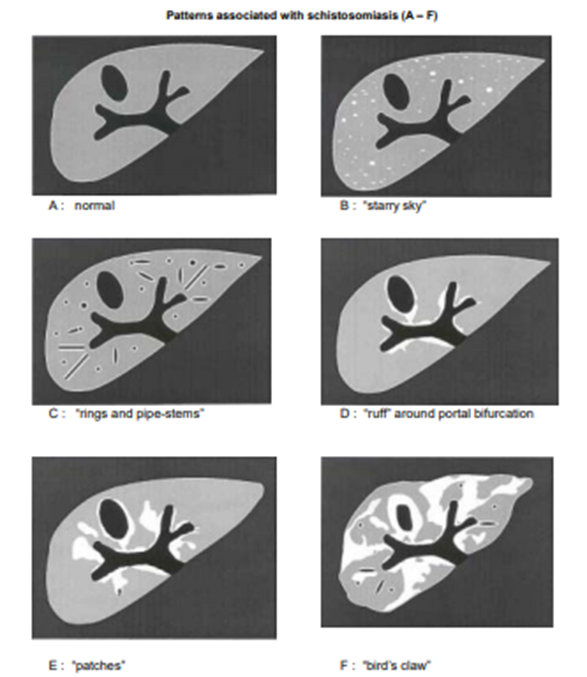

Supplement: S1 Fig — (TIF) [file pntd.0009375.s001.tif]
