# Supplementary material for: Morbidity associated with Schistosoma mansoni infection in north-eastern Democratic Republic of the Congo
Source: PLoS Negl Trop Dis. 2021 Dec 2;15(12):e0009375. doi: 10.1371/journal.pntd.0009375 (PMC8638987; doi:10.1371/journal.pntd.0009375)
Supplement: S1 Table — Study conducted in 13 purposively selected villages in Ituri province (n = 586). Only diagnostic results of Kato-Katz (KK) tests have been considered. (DOCX) [file pntd.0009375.s002.docx]

**S1 Table:** **Univariable associations with *S. mansoni* infection in the 2017 morbidity study based on Kato-Katz test only.** Study conducted in 13 purposively selected villages in Ituri province (n=586). Only results of Kato-Katz (KK) diagnostic approach have been considered.

Characteristics *S. mansoni* (+) *S. mansoni* (-)

N=347 N=239

n % n % OR (95% CI) p-value

Gender*

Females 190 55.6 152 44.4 1.0

Males 157 64.3 87 35.7 1.44 (1.03–2.03) 0.033

Age categories (years) *

6 – 9 73 59.4 50 40.6 1.0

10 – 14 91 65.0 49 35.0 1.27 (0.77–2.10) 0.346

15 – 19 48 71.6 19 28.4 1.73 (0.91–3.30) 0.093

20 – 29 50 64.9 27 35.1 1.27 (0.70–2.29) 0.431

30 – 39 37 54.4 31 45.6 0.81 (0.45–1.49) 0.510

40 – 49 25 48.1 27 51.9 0.63 (0.33–1.22) 0.171

≥50 23 39.0 36 61.0 0.44 (0.23–0.84) 0.010

STH

*T. trichiura* (Y/N) 1 0.3 2 0.84 0.34 (0.03–3.81) 0.361

*A. lumbricoides* (Y/N) 1 0.3 0 0.0 na

Hookworm (Y/N) * 4 0.3 3 1.3 0.23 (0.02–2.21) 0.163

Anthropometry (BMI)*

Obese (Y/N) 25 7.2 33 13.8 1.0

Overweight (Y/N) 11 3.2 13 5.4 1.12 (0.42–3.92) 0.822

Normal weight (Y/N) 140 40.4 96 40.2 1.92 (1.07–3.46) 0.026

Underweight (Y/N) 171 49.3 97 40.6 2.33 (1.30–4.18) 0.004

Clinical findings

Diarrhoea (Y/N) * 96 27.7 41 17.2 1.85 (1.22–2.79) 0.003

Blood in stool (Y/N) * 84 24.2 42 17.6 1.50 (0.99–2.27) 0.055

Abdominal pain (Y/N) 185 53.3 124 51.9 1.06 (0.76–1.47) 0.733

Hematemesis (Y/N) 2 0.6 3 1.3 0.46 (0.08–2.76) 0.380

Ultrasound findings

Hepatomegaly (Y/N) * 101 29.1 54 22.6 1.41 (0.96–2.06) 0.079

Splenomegaly (Y/N) * 100 28.8 48 20.1 1.61 (1.09–2.39) 0.017

Ascites (Y/N) * 1 0.3 3 1.3 0.23 (0.02–2.21) 0.163

A/B patterns (Y/N) * 182 52.5 146 61.1 1.0

C/D patterns (Y/N) 133 38.3 80 33.5 1.33 (0.94–1.95) 0.109

E/F patterns (Y/N) 28 8.1 10 4.2 2.25 (1.05–4.80) 0.032

Fatty liver (Y/N) 2 0.8 4 1.2 1.38 (0.25–7.62) 0.709

Other (Y/N) 1 0.4 0 0.0 na

* Included in the multivariable analysis. BMI, body mass index; na, not applicable; A pattern: normal; B pattern: “starry sky”; C pattern: “rings and pipe-stems”; D pattern “highly echogenic ruff around portal bifurcation”; E pattern “highly echogenic patches”; F pattern: “highly echogenic bands and streaks – bird’s claw”; Fatty liver (Y pattern) and other abnormality (Z pattern) indicate pathology different from periportal fibrosis [1, 2].

References

1. WHO. ULTRASOUND IN SCHISTOSOMIASIS. A Practical Guide to the Standarized Use of Ultrasonography for the Assessment of Schistosomiasis-related Morbidity. World Health Library. 2000.

2. Richter J, Domingues ALC, Barata CH, Prata AR, Lambertucci JR. Report of the second satellite symposium on ultrasound in schistosomiasis. Mem I Oswaldo Cruz. 2001;96:151-6. doi: Doi 10.1590/S0074-02762001000900023.
