# Supplementary material for: Morbidity associated with Schistosoma mansoni infection in north-eastern Democratic Republic of the Congo
Source: PLoS Negl Trop Dis. 2021 Dec 2;15(12):e0009375. doi: 10.1371/journal.pntd.0009375 (PMC8638987; doi:10.1371/journal.pntd.0009375)
Supplement: S2 Table — Results of the multivariable analysis of risk factors for morbidity due to Schistosoma mansoni infection among participants from 13 villages in Ituri province (n = 586). Only diagnostic results of Kato-Katz (KK) tests have been considered. (DOCX) [file pntd.0009375.s003.docx]

**S2 Table. Risk factors for morbidity due to *Schistosoma mansoni* infection, 2017 study.** Results of the multivariable analysis of risk factors for morbidity due to *Schistosoma mansoni* infection among participants from 13 villages in Ituri province (n=586). Only results of Kato-Katz (KK) diagnostic approach have been considered.

Risk factors aOR (95% CI) Std. Err. z p-value

Demographic risk factors

Age 0.98 (0.97–0.99) 0.006 -3.48 <0.001

Gender (Male/Female) 1.43 (1.00–2.12) 0.279 1.94 0.052

Anthropometric risk factors

BMI 1.00 (0.95–1.04) 0.024 -0.18 0.857

Clinical finding

Diarrhoea 1.76 (1.12–2.74) 0.400 2.47 0.013

Blood in stool 1.13 (0.26–1.77) 0.261 0.52 0.606

Ultrasound findings

Hepatomegaly (Yes/No) 1.52 (0.99–2.32) 0.329 1.93 0.053

Splenomegaly (Yes/No) 1.12 (0.73–1.72) 0.246 0.50 0.619

Ascites (Yes/No) 0.13 (0.01–1.37) 0.156 -1.70 0.089

Liver pathology (Yes/No) 1.20 (1.06–1.37) 0.080 2.80 0.005

Co-infection

Hookworm (Yes/No) 0.20 (0.02–2.06) 0.240 -1.35 0.177

aOR: adjusted odds ratio; CI: confidence interval. BMI, body mass index (only taken as continious variable).
