# Supplementary material for: Morbidity associated with Schistosoma mansoni infection in north-eastern Democratic Republic of the Congo
Source: PLoS Negl Trop Dis. 2021 Dec 2;15(12):e0009375. doi: 10.1371/journal.pntd.0009375 (PMC8638987; doi:10.1371/journal.pntd.0009375)
Supplement: S3 Table — Study conducted in 13 purposively selected villages in Ituri province (n = 586). Only diagnostic results of the point-of-care circulating cathodic antigen (POC-CCA) tests have been considered. (DOCX) [file pntd.0009375.s004.docx]

**S3 Table: Morbidity association with *S. mansoni* infection in the 2017 study based on POC-CCA diagnostic approach.** Results of the univariable analysis of data from 13 purposively selected villages of Ituri province (n=586).

Characteristics *S. mansoni* (+) *S. mansoni* (-)

N=385 N=201

n % n % OR (95% CI) p-value

Gender*

Females 222 57.7 120 59.7 1.0

Males 163 42.3 81 40.3 1.09 (0.77-1.54) 0.635

Age categories (years)*

6 – 9 80 20.8 43 21.4 1.0

10 – 14 105 27.3 35 17.4 1.61 (0.94-2.76) 0.078

15 – 19 45 11.7 22 11.0 1.10 (0.58-2.07) 0.769

20 – 29 57 14.8 20 10.0 1.53 (0.81-2.89) 0.184

30 – 39 40 10.4 28 14.0 0.77 (0.42-1.42) 0.396

40 – 49 30 7.8 22 11.0 0.73 (0.38-1.43) 0.359

≥50 28 7.3 31 15.4 0.49 (0.26-0.92) 0.024

STH

*T. trichiura* (Y/N) 1 0.3 2 1.0 0.26 (0.02-2.89) 0.237

*A. lumbricoides* (Y/N) 1 0.3 0 0.0 na

Hookworm (Y/N) * 1 0.3 3 1.5 0.17 (0.02-1.68) 0.086

Anthropometry (BMI)*

Obese (Y/N) 29 7.5 29 14.4 1.0

Overweight (Y/N) 17 4.4 7 3.5 2.43 (0.85-6.91) 0.086

Normal weight (Y/N) 155 40.3 81 40.3 1.91 (1.06-3.44) 0.027

Underweight (Y/N) 184 47.8 84 41.8 2.19 (1.22-3.93) 0.007

Clinical findings

Diarrhoea (Y/N) * 98 25.5 39 19.4 1.42 (0.93-2.16) 0.101

Blood in stool (Y/N) 91 23.6 35 17.4 1.47 (0.95-2.27) 0.082

Abdominal pain (Y/N) 203 52.7 106 52.7 1.00 (0.71-1.41) 0.998

Hematemesis (Y/N) 4 1.0 1 0.5 2.10 (0.23-18.96) 0.499

Ultrasound findings

Hepatomegaly (Y/N) * 109 28.3 46 22.9 1.33 (0.89-1.98) 0.158

Splenomegaly (Y/N) * 101 26.2 47 23.4 1.17 (0.78-1.74) 0.451

Ascites (Y/N) * 2 0.5 2 1.0 0.52 (0.07-3.73) 0.507

A/B patterns (Y/N) * 211 54.8 117 58.2 1.0

C/D patterns (Y/N) 145 37.7 68 33.8 1.18 (0.82–1.71) 0.370

E/F patterns (Y/N) 26 6.8 12 6.0 1.20 (0.58–2.47) 0.618

Fatty liver (Y/N) 4 2.0 2 0.5 0.26 (0.05–1.42) 0.094

Other (Y/N) 0 0.0 1 0.3 na

* Included in the multivariable analysis. BMI, body mass index; na, not applicable; A pattern: normal; B pattern: “starry sky”; C pattern: “rings and pipe-stems”; D pattern “highly echogenic ruff around portal bifurcation”; E pattern “highly echogenic patches”; F pattern: “highly echogenic bands and streaks – bird’s claw”; Fatty liver (Y pattern) and other abnormality (Z pattern) indicate pathology different from periportal fibrosis [1, 2].

References

1. WHO. ULTRASOUND IN SCHISTOSOMIASIS. A Practical Guide to the Standarized Use of Ultrasonography for the Assessment of Schistosomiasis-related Morbidity. World Health Library. 2000.

2. Richter J, Domingues ALC, Barata CH, Prata AR, Lambertucci JR. Report of the second satellite symposium on ultrasound in schistosomiasis. Mem I Oswaldo Cruz. 2001;96:151-6. doi: Doi 10.1590/S0074-02762001000900023.
