# Supplementary material for: Morbidity associated with Schistosoma mansoni infection in north-eastern Democratic Republic of the Congo
Source: PLoS Negl Trop Dis. 2021 Dec 2;15(12):e0009375. doi: 10.1371/journal.pntd.0009375 (PMC8638987; doi:10.1371/journal.pntd.0009375)
Supplement: S4 Table — Results of the multivariable analysis of risk factors for morbidity due to Schistosoma mansoni infection among participants from 13 villages in Ituri province (n = 586). Only results of the point-of-care circulating cathodic antigen (POC-CCA) diagnostic tests have been considered. (DOCX) [file pntd.0009375.s005.docx]

**S4 Table. Risk factors for morbidity due to *Schistosoma mansoni* infection, 2017 study.** Results of the multivariable analysis of risk factors for morbidity due to *Schistosoma mansoni* infection among participants from 13 villages in Ituri province (n=586). Results of POC-CCA diagnostic approach have been considered.

Risk factors aOR (95% CI) Std. Err. z p-value

Demographic risk factors

Age 0.98 (0.97–0.99) 0.006 -3.69 <0.001

Gender (Male/Female) 1.07 (0.73–1.57) 0.208 0.35 0.727

Anthropometric risk factors

BMI 1.01 (0.96–1.07) 0.025 0.30 0.762

Clinical finding

Diarrhoea 1.29 (0.83–2.03) 0.296 1.13 0.260

Blood in stool 1.31 (0.83–2.10) 0.313 1.15 0.250

Ultrasound findings

Hepatomegaly (Yes/No) 1.55 (1.01–2.40) 0.345 1.99 0.046

Splenomegaly (Yes/No) 0.84 (0.54–1.29) 0.185 -0.80 0.423

Ascites (Yes/No) 0.56 (0.07–4.26) 0.579 -0.56 0.573

Liver pathology (Yes/No) 1.04 (0.91–1.18) 0.068 0.54 0.589

Co-infection

Hookworm (Yes/No) 0.15 (0.01–1.50) 0.174 -1.62 0.106

aOR: adjusted odds ratio; CI: confidence interval. BMI, body mass index (only taken as continious variable).
