# Supplementary material for: Morbidity associated with Schistosoma mansoni infection in north-eastern Democratic Republic of the Congo
Source: PLoS Negl Trop Dis. 2021 Dec 2;15(12):e0009375. doi: 10.1371/journal.pntd.0009375 (PMC8638987; doi:10.1371/journal.pntd.0009375)
Supplement: S5 Table — Results from 13 purposively selected villages of Ituri province (n = 586). Prevalence derived from combined diagnostic approach and intensity determined by Kato-Katz test results. (DOCX) [file pntd.0009375.s006.docx]

**S5 Table: Prevalence of periportal fibrosis (PPF) by age, sex, village, and *S. mansoni* infection status in the 2017 study.** Results from 13 purposively selected villages of Ituri province (n=586). Prevalence with the combined diagnostic approach and intensity with Kato-Katz diagnostic approach.

Characteristics Periportal fibrosis (PPF) *S. mansoni* infection status____________________________________ __________________________________ Prevalence ____________ Intensity*

Overall (%) Female (%) Male (%) Overall (%) Female (%) Male (%) Overall Female Male

Overall 251 (42.8) 147 (43.0) 104 (42.6) 449 (76.6) 257 (75.2) 192 (78.7) 109.7 91.1 135.8

Age categories

6 – 9 46 (37.4) 26 (41.3) 20 (33.3) 92 (74.8) 45 (71.4) 47 (78.3) 112.6 57.0 170.9

10 – 14 52 (37.1) 29 (40.9) 23 (33.3) 119 (85.0) 60 (84.5) 59 (85.5) 156.1 131.1 181.8

15 – 19 34 (50.8) 17 (44.7) 17 (58.6) 54 (80.6) 28 (73.7) 26 (89.7) 133.9 140.8 124.9

20 – 29 40 (52.0) 33 (52.4) 7 (50.0) 63 (81.8) 50 (79.4) 13 (92.9) 110.6 111.9 104.9

30 – 39 29 (42.7) 17 (37.8) 12 (52.2) 48 (70.6) 31 (68.9) 17 (73.9) 76.3 94.7 40.4

40 – 49 21 (40.4) 12 (37.5) 9 (45.0) 35 (67.3) 22 (68.8) 13 (65.0) 85.2 18.8 191.4

≥50 29 (49.2) 13 (43.3) 16 (55.2) 38 (64.4) 21 (70.0) 17 (58.6) 25.1 32.7 17.1

Villages

Bankoko 8 (29.6) 3 (20.0) 5 (41.7) 9 (33.3) 3 (20.0) 6 (50.0) 3.2 0.3 6.8

Lumumba 5 (22.7) 3 (20.0) 2 (28.6) 14 (63.4) 11 (73.3) 3 (42.9) 60.0 57.7 64.9

Simbilyabo 14 (22.2) 6 (15.4) 8 (33.3) 35 (55.6) 23 (59.0) 12 (50.0) 46.1 48.3 42.6

Mangenengene 20 (54.1) 12 (54.6) 8 (53.3) 26 (70.3) 16 (72.7) 10 (66.7) 14.6 10.6 20.5

Kadjugi 23 (37.1) 14 (35.9) 9 (39.1) 45 (72.6) 26 (66.7) 19 (82.6) 94.3 29.7 203.8

Kindia 8 (12.7) 7 (21.2) 1 (3.3) 52 (82.5) 26 (78.8) 26 (86.7) 30.7 38.0 22.7

Gupe 65 (63.7) 33 (57.9) 32 (71.1) 79 (77.5) 44 (77.2) 35 (77.8) 97.3 124.2 63.3

Sukisa 11 (26.2) 10 (38.5) 1 (6.3) 33 (78.6) 21 (80.8) 12 (75.0) 29.4 20.2 44.4

Ngezi 33 (54.1) 23 (65.7) 10 (38.5) 53 (86.9) 28 (80.0) 25 (96.2) 102.5 65.9 151.9

Mambau 8 (47.1) 6 (46.2) 2 (50.0) 16 (94.1) 13 (100) 3 (75.0) 193.4 249.6 11.1

Mandima 25 (62.5) 17 (60.7) 8 (66.7) 39 (97.5) 27 (96.4) 12 (100) 150.9 156.6 137.6

Pekele 26 (60.5) 11 (64.7) 15 (57.7) 41 (95.4) 16 (94.1) 25 (96.2) 519.3 459.1 558.7

Ndaru-Muswa 5 (71.4) 2 (66.7) 3 (75.0) 7 (100) 3 (100) 4 (100) 370.3 136.0 546.0

*Note: Intensity = arithmetic mean egg peg gram (EPG) count.
