# Supplementary material for: Morbidity associated with Schistosoma mansoni infection in north-eastern Democratic Republic of the Congo
Source: PLoS Negl Trop Dis. 2021 Dec 2;15(12):e0009375. doi: 10.1371/journal.pntd.0009375 (PMC8638987; doi:10.1371/journal.pntd.0009375)
Supplement: S6 Table — Results from 13 purposively selected villages in Ituri province (n = 586). Prevalence derived from a combined diagnostic approach. (DOCX) [file pntd.0009375.s007.docx]

**S6 Table: Prevalence of hepatomegaly, splenomegaly and overall organomegaly by age, sex, village, and *S. mansoni* infection status in the 2017 study.** Results from 13 purposively selected villages of Ituri province (n=586). Prevalence with the combined diagnostic approach.

Characteristics No Organ enlargement status ____ *S. mansoni* infection status *Organomegaly Hepatomegaly Splenomegaly *Hepatosplenomegaly Prevalence Intensity

n % n % n % n % n % mean EPG

_______________________________________________________________________________________________________________________

Overall 586 254 (43.3) 155 (26.5) 148 (25.3) 49 (8.4) 449 (76.6) 109.7

Sex

Female 342 131 (38.3) 75 (21.9) 85 (24.9) 29 (8.5) 257 (75.2) 91.1

Male 244 123 (50.4) 80 (32.8) 63 (25.8) 20 (8.2) 192 (78.7) 135.8

Age categories

6 – 9 123 60 (48.8) 23 (18.7) 50 (40.7) 13 (10.6) 47 (78.3) 112.6

10 – 14 140 44 (31.4) 13 (9.3) 40 (28.6) 9 (6.4) 59 (85.5) 156.1

15 – 19 67 40 (59.7) 31 (46.3) 20 (29.9) 11 (16.4) 26 (89.7) 133.9

20 – 29 77 35 (45.5) 24 (31.2) 17 (22.1) 6 (7.8) 13 (92.9) 110.6

30 – 39 68 30 (44.1) 27 (39.7) 10 (14.7) 7 (10.3) 17 (73.9) 76.3

40 – 49 52 22 (42.3) 17 (32.7) 7 (13.5) 2 (3.9) 13 (65.0) 85.2

≥50 59 23 (39.0) 20 (33.9) 4 (6.8) 1 (1.7) 17 (58.6) 25.1

Villages

Bankoko 27 10 (37.0) 8 (29.6) 6 (22.2) 4 (14.8) 6 (50.0) 3.2

Lumumba 22 5 (22.7) 0 (0.0) 5 (22.7) 0 (0.0) 3 (42.9) 60.0

Simbilyabo 63 19 (30.2) 12 (19.1) 8 (12.7) 1 (1.6) 12 (50.0) 46.1

Mangenengene37 22 (59.5) 8 (21.6) 17 (46.0) 3 (8.1) 10 (66.7) 14.6

Kadjugi 62 24 (38.7) 19 (30.7) 8 (12.9) 3 (4.8) 19 (82.6) 94.3

Kindia 63 17 (27.0) 15 (23.8) 5 (7.9) 3 (4.8) 26 (86.7) 30.7

Gupe 102 47 (46.1) 27 (26.5) 25 (24.5) 5 (4.9) 35 (77.8) 97.3

Sukisa 42 14 (33.3) 12 (28.6) 4 (9.5) 2 (4.8) 12 (75.0) 29.4

Ngezi 61 22 (36.1) 14 (23.0) 11 (18.0) 3 (4.9) 25 (96.2) 102.5

Mambau 17 13 (76.5) 9 (52.9) 9 (52.9) 5 (29.4) 3 (75.0) 193.4

Mandima 40 27 (67.5) 11 (27.5) 23 (57.5) 7 (17.5) 12 (100) 150.9

Pekele 43 28 (65.1) 15 (34.9) 21 (48.8) 8 (18.6) 25 (96.2) 519.3

Ndaru-Muswa 7 6 (85.7) 5 (71.4) 6 (85.7) 5 (71.4) 4 (100) 370.3

* Organomegaly = either hepatomegaly or splenomegaly; hepatosplenomegaly = simultaneous hepatomegaly and splenomegaly
