# Supplementary material for: Morbidity associated with Schistosoma mansoni infection in north-eastern Democratic Republic of the Congo
Source: PLoS Negl Trop Dis. 2021 Dec 2;15(12):e0009375. doi: 10.1371/journal.pntd.0009375 (PMC8638987; doi:10.1371/journal.pntd.0009375)
Supplement: S7 Table — Study conducted in 13 purposively selected villages in Ituri province (n = 586). Only results of the Kato-Katz diagnostic tests have been considered in this analysis. (DOCX) [file pntd.0009375.s008.docx]

**S7 Table: Liver pattern prevalence and association with *S. mansoni* infection intensity in the 2017 study.** Study conducted in 13 purposively selected villages in Ituri province (n=586). Results of the Kato-Katz diagnostic approach have been considered in this analysis.

Characteristics ________________*S. mansoni* infection intensity________

Number (%) Negative Light Moderate Heavy

n % n % n % n % χ² p-value

Overall 586 (100) 239 40.8 216 36.9 89 15.2 42 7.2

Liver patterns

A pattern 267 (45.6) 124 46.4 92 34.5 36 13.5 15 5.6

B pattern 61 (10.4) 22 36.1 28 45.9 7 11.5 4 6.6

C pattern 156 (26.6) 61 39.1 56 35.9 26 16.7 13 8.3

D pattern 57 (9.7) 19 33.3 23 40.4 11 19.3 4 7.2

E pattern 23 (3.9) 3 13.4 8 34.8 8 34.8 4 17.4

F pattern 15 (2.6) 7 46.7 5 33.3 1 6.7 2 13.3

Fatty liver 6 (1.0) 2 33.3 4 66.7 0 0.0 0 0.0

Other 1 (0.4) 1 100 0 0.0 0 0.0 0 0.0 28.01 0.140

A pattern: normal; B pattern: “starry sky”; C pattern: “rings and pipe-stems”; D pattern “highly echogenic ruff around portal bifurcation”; E pattern “highly echogenic patches”; F pattern: “highly echogenic bands and streaks – bird’s claw”; Fatty liver (Y pattern) and other abnormality (Z pattern) indicate pathology different from periportal fibrosis [1, 2].

Reference

1. WHO. ULTRASOUND IN SCHISTOSOMIASIS. A Practical Guide to the Standarized Use of Ultrasonography for the Assessment of Schistosomiasis-related Morbidity. World Health Library. 2000.

2. Richter J, Domingues ALC, Barata CH, Prata AR, Lambertucci JR. Report of the second satellite symposium on ultrasound in schistosomiasis. Mem I Oswaldo Cruz. 2001;96:151-6. doi: Doi 10.1590/S0074-02762001000900023.
