# Supplementary material for: Morbidity associated with Schistosoma mansoni infection in north-eastern Democratic Republic of the Congo
Source: PLoS Negl Trop Dis. 2021 Dec 2;15(12):e0009375. doi: 10.1371/journal.pntd.0009375 (PMC8638987; doi:10.1371/journal.pntd.0009375)
Supplement: S8 Table — Results from 13 purposively selected villages of Ituri province (n = 586). Analysis of Kato-Katz results, POC-CCA results, and the combined diagnostic approach results. (DOCX) [file pntd.0009375.s009.docx]

**S8 Table: Univariable analysis of risk of liver patterns and periportal fibrosis (PPF) by *S. mansoni* infection status with different diagnostic approaches in the 2017 study.** Results from 13 purposively selected villages of Ituri province (n=586). Analysis with Kato-Katz diagnostic approach, POC-CCA diagnostic approach, and with the combined diagnostic approach.

Liver patterns/ Kato-Katz ______ POC-CCA _____ Kato-Katz+POC-CCA

PPF OR (95% CI) p-value OR (95% CI) p-value OR (95% CI) p-value

A pattern 0.65 (0.47-0.91) 0.011 0.99 (0.70-1.39) 0.942 0.77 (0.53-1.14) 0.198

B pattern 1.25 (0.72-2.17) 0.429 0.73 (0.42-1.25) 0.246 1.14 (0.60-2.18) 0.687

C pattern 1.10 (0.76-1.60) 0.618 1.02 (0.69-1.50) 0.920 1.08 (0.69-1.66) 0.746

D pattern 1.42 (0.80-2.54) 0.229 1.52 (0.82-2.82) 0.182 1.31 (0.66-2.60) 0.444

E pattern 4.81 (1.40-16.53) 0.006 2.56 (0.85-7.65) 0.082 3.31 (0.76-14.38) 0.090

F pattern 0.78 (0.28-2.19) 0.639 0.45 (0.16-1.25) 0.116 0.45 (0.16-1.28) 0.124

Fatty liver 1.73 (0.31-9.67) 0.525 0.26 (0.05-1.48) 0.103 1.74 (0.20-15.25) 0.611

Other . 0.285 0.470 0.556

PPF categories

Normal (A/B) 0.70 (0.50-0.98) 0.039 0.87 (0.62-1.23) 0.431 0.81 (0.55-1.20) 0.296

Mild (C/D) 1.23 (0.87-1.74) 0.230 1.18 (0.83-1.69) 0.360 1.17 (0.78-1.75) 0.441

Severe (E/F) 2.01 (0.95-4.23) 0.061 1.14 (0.56-2.31) 0.715 1.15 (0.52-2.58) 0.726

Overall PPF

PPF (Yes/No) 1.43 (1.02-2.01) 0.036 1.21 (0.85-1.71) 0.284 1.20 (0.81-1.78) 0.356

A pattern: normal; B pattern: “starry sky”; C pattern: “rings and pipe-stems”; D pattern “highly echogenic ruff around portal bifurcation”; E pattern “highly echogenic patches”; F pattern: “highly echogenic bands and streaks – bird’s claw”; Fatty liver (Y pattern) and other abnormality (Z pattern) indicate pathology different from periportal fibrosis [1, 2].

References

1. WHO. ULTRASOUND IN SCHISTOSOMIASIS. A Practical Guide to the Standarized Use of Ultrasonography for the Assessment of Schistosomiasis-related Morbidity. World Health Library. 2000.

2. Richter J, Domingues ALC, Barata CH, Prata AR, Lambertucci JR. Report of the second satellite symposium on ultrasound in schistosomiasis. Mem I Oswaldo Cruz. 2001;96:151-6. doi: Doi 10.1590/S0074-02762001000900023.
